# Intersectional experiences of non-communicable diseases and health seeking strategies in informal settlements in Freetown, Sierra Leone

**DOI:** 10.1371/journal.pgph.0005263

**Published:** 2026-07-01

**Authors:** Abu Conteh, Laura Dean, Annie Wilkinson, Joseph Macarthy, Braima Koroma, Sally Theobald

**Affiliations:** 1 Sierra Leone Urban Research Centre, Freetown, Sierra Leone; 2 Liverpool School of Tropical Medicine, Department of International Public Health, Pembroke Place, Liverpool, United Kingdom; 3 Institute of Development Studies, Brighton, United Kingdom; PLOS: Public Library of Science, UNITED STATES OF AMERICA

## Abstract

This paper takes an intersectional approach to explore the burdens of non-communicable diseases (NCDs) and their interactions with gendered inequities and poverty, in shaping health seeking practices. The study explores the lived experiences of men and women living with NCDs in three informal settlements in Freetown, Sierra Leone. As Low- and Middle-Income Countries (LMICs) grapple with increased NCD risk factors, evidence gaps limit understanding of the complex ways in which gendered vulnerabilities are shaped. This study provides insights into the multiple axes of inequity which shape NCD outcomes and healthcare access barriers and suggests ways to improve NCD services. This study therefore adapts and applies the Intersectional Gender Analysis Framework for Infectious Diseases of Poverty in an informal settlement context to understand how NCDs impact men and women differently and influence different health seeking and treatment outcomes. We conducted narrative interviews with 15 participants living with diabetes, hypertension, and disability related to stroke through three household visits for a period of 12 weeks. Findings highlight lived experience of NDCs, coping strategies and healthcare seeking patterns, influenced by poverty, gender inequities, and NCD comorbidities. Women’s lived experiences of NCDs reflect historical and patriarchal disadvantages through their limited financial autonomy, barriers to healthcare decision making and treatment access, compounded by gendered impacts of conflict and migration. Men’s experiences included an erosion of social status and financial autonomy, limiting access to healthcare and coping. Gender differences and patriarchal norms influenced household and healthcare decision making, reflecting the division of roles and access to resources by men and women, which shaped different care seeking pathways and treatment outcomes. Our study shows that the burden of NCDs and healthcare decision making are influenced by intersecting structural barriers, which requires that NCD healthcare must be approached from bio-social, rather than solely a biomedical perspective.

## Introduction

Globally, NCDs constitute a significant public health threat, accounting for about 73% of all deaths [1,2], WHO reported that of the 43 million global deaths resulting from NCDs, 73% affect LMIC settings (WHO, 2024) [3]. Among the growing burden of NCDs, cardiovascular diseases (CVDs) pose the greatest threat of NCD related mortality. WHO estimates that CVDs alone cause over 17 million deaths annually, over three quarters of which occur in LMICs.

As LMICs face epidemiological transitions [4], people living with NCDs in urban informal settlements are impacted by underlying structural drivers, such as limited access to healthcare services, inadequate housing, and persistent health inequities. Sierra Leone is no exception to these trends. For example, over 70% adults in Sierra Leone are exposed to NCD risks due to limited access to healthy diets, tobacco and alcohol abuse, environmental pollution and economic precarity. These factors contribute to about 30,000 deaths annually [5]. Among the top ten causes of death in Sierra Leone, NCDs such as ischemic heart diseases and stroke are ranked 4^th^ and 6^th^ respectively [6].

However, the interactions between the multiple layers of vulnerability, such as poverty, gender, healthcare and housing inequities in marginalised urban settlements and NCD outcomes remain poorly understood and are often not prioritised within health systems [7, 8]. This paper therefore takes an intersectional approach to explore the burdens of NCDs and health seeking among people living with diabetes, hypertension and disability resulting from stroke in three informal settlements in Freetown, Sierra Leone. Intersectional approaches are critical in understanding health inequities, and in shaping how women and men cope with health crises, including chronic diseases [9, 10]. Applying intersectionality in health systems research will help policy makers in fragile health system contexts better understand diverse ways to tackle health inequities and support progress towards Universal Health Coverage by 2030 [11].

Within LMICs, rapid urbanisation is of relevance to rising NCDs, particularly in the expansion of informal settlements. These areas face compounded risks, driven by inequitable urban policies and planning processes that exclude residents from essential services due to housing and tenure insecurity [12], further exposing them to adverse health conditions [13]. Urbanisation has also strained health systems, resulting in limited capacity to provide protection and care for the urban poor [8]. Without understanding and addressing these drivers, countries will struggle to address their NCD burdens sustainably or equitably.

In Sierra, Leone, informal settlement residents face health precarities because of everyday struggles with poor housing, financial stress, limited healthcare, and environmental hazards, which intensify the burdens of chronic diseases, particularly during health shocks such as the COVID-19 outbreak [13, 14]. A study conducted in an informal settlement in Freetown - Kroobay, identified diabetes and hypertension as risk factors linked to poor diets as a result of poverty, limited physical activity as a result of livelihood commitments, environmental pollution, and stress about housing challenges [15]. Social and environmental risk factors such as smoke pollution, financial stress, poor access to housing, and limited access to clean drinking water and healthy diets are commonplace in informal settlements in Freetown [13]. NCDs, interacting with poverty and other forms of social inequity can therefore be particularly detrimental to people living in informal settlements. As Elsey et al., [8] write, addressing urban health inequalities, particularly in the face of rising NCD threats requires a rethink of broader systemic drivers shaping these risks among the urban poor.

A further axis of health disadvantage is gender inequity. The creation of gender-blind health policies and interventions in fragile settings signals that the healthcare needs of women and men affected by NCDs are not always met [16]. Morgan et al., [17] noted that gender-blind health policies have wider consequences beyond health outcomes, citing that the lack of such considerations negatively impacted the wellbeing of women and men during the COVID-19 outbreak, in marginalised urban spaces [17]. Therefore, gender gaps need addressing in the planning and delivery of healthcare, as gender roles and norms often shape how women and men experience and live with health crisis, and in navigating the barriers to healthcare [18]. However, significant gaps still exist in understanding these intersectional drivers of NCD vulnerabilities in Sierra Leone.

### Gender, health and other axes of inequity in Sierra Leone

Gender disparities in Sierra Leone underlie women’s barriers to healthcare, educational and livelihood opportunities. National statistics show that women are significantly disadvantaged with respect to educational attainment, access to healthcare, financial services and the labour market. For example, literacy rates among women (43%) are far lower than men (62%). Additionally, gender disparities in wage earnings are huge as only 45% of employed women receive financial reward for their work, compared to 72% of men [19]. These structural barriers reflect women’s lack of autonomy in household and healthcare decision making, including sexual and reproductive health rights [20]. A gender and intersectional analysis is vital in understanding the drivers of health inequities and in enhancing an inclusive and integrated healthcare delivery system that meets the needs of marginalised urban residents.

Previous research into health in informal settlements indicate that gender roles, with respect to the management of long-term illness and health seeking are reinforced by socio-cultural norms [13]. Societal expectations of women firmly place caregiving roles into their hands, while decision making rights (including healthcare) are accorded to men, which negatively impact women’s health outcomes and access to care [14]. A further look into gendered health inequities in Freetown’s informal settlements found that women’s uptake of COVID-19 vaccination and related healthcare services, (e.g., reproductive health services) were hindered by livelihood, and care responsibilities, influenced by historical barriers to education and the lack of inclusion in household decision making [21]. These experiences of access to preventive medicine, including COVID-19 vaccines were different for men as they were more likely to be formally employed, which created more incentive for them to be vaccinated through regulations on the mandatory possession of vaccine cards to access public buildings [21].

While women experience these gendered health barriers, men also face health crisis and their own challenges. Men’s experiences of health crisis are shaped, not only by physical manifestations of disease, but by changes in social status because of loss of financial autonomy and respect [22]. While these studies focused on the intersections of gender and health, they did not explicitly focus on gender asymmetries as a driver of NCDs outcomes. This study therefore builds on current understandings of gender hierarchies and their interactions with broader structural drivers such as poverty in shaping the experiences of NCD outcomes and health seeking strategies. An intersectional analysis approach was applied, informed by the Intersectional Gender Analysis Framework for Infectious Diseases of Poverty Research [3] through participants’ narratives of NCD outcomes and health seeking decision making. This study therefore takes an intersectional approach to explore the burdens of NCDs and their interactions with structural barriers including gender inequities, poverty and health seeking practices.

## Materials and methods

### Ethics statement

Ethics approvals were sought and granted by the Sierra Leone Ethics and Scientific Review Committee (SLESRC) on 3^rd^ of August 2021 (Version 2.0 of 28 July 2021). Approval was also granted by the Research and Ethics Committee of the Liverpool School of Tropical Medicine on 5^th^ August 2021 with reference number 21-043.

### Study setting and link within broader ARISE research programme

This study was conducted in three informal settlements in Freetown which face challenges with service delivery, including access to water, sanitation, and healthcare. Our research sites included Cockle Bay, which is a seaside settlement in the west of Freetown, and Dwarzark and Moyiba which are both hillside settlements in the central and far east of the city. The settlements were purposively selected to ensure diverse voices in informal settlements, and to explore how their experiences of the places they live in shape their health experiences and treatment seeking. Our approach to understand the peculiarities of informal settlements, despite the similar challenges they face, is important in designing health programmes particularly for people with long term illnesses. The study was undertaken within the Accountability and Equity in Informal Settlements (ARISE) which worked with informal settlement residents including co-researchers (people living and working in informal settlements) in addressing intractable health challenges [23]. This working relationship aimed at strengthening equitable partnerships with marginalised urban resident, through active participation in research design and data collection [24], to improve health and wellbeing outcomes. Both studies had similar understanding of the drivers of health inequities, although their focus and methodological approaches varied. The wider ARISE study employed multi-staged participatory and mixed methods approaches to understand health inequities from a governance and accountability perspective, while this study employed an intersectional lens, drawing on participants’ health narratives. The participants were selected independent of the ARISE research.

### Methodology, methods and participants

We conducted narrative interviews to explore the lived experiences of men and women living with NCD health conditions and health seeking priorities, each lasting over an hour. Narrative interviews form a part of the narrative inquiry methodology which allows participants to reflect on their lived experiences, within broader social, political and environmental contexts [25]. The interviews were conducted through three household visits to a cohort of 15 participants living with diabetes, hypertension, and disability resulting from stroke for a period of 12 weeks (S1 Text). The interviews were divided into two phases: the first part involved life histories, exploring participants’ narratives about ill health, livelihood and educational attainment and household gender relations. The second part included two rounds of interviews exploring health seeking decisions, including health beliefs, cost of care and quality of care, using visual diaries to represent the different providers they had visited in the last one month prior to the interview session. The visual diaries were designed to help the participants to recall their health seeking journeys and to start a conversation about their preferred providers, the type of conditions presented and the treatment outcomes from the different providers (S1 Table). The diaries were also used as a guide for follow up questions, and to plan the next visit. All interviews were audio recorded following consent procedures.

### Recruitment of participants

We designed a purposive sampling strategy to identify and recruit participants for the narrative interviews (S2 Text). Participant selection was based on predetermined criteria, including type of NCD, age, gender, and place of living within the settlements to achieve a diversity of participants’ characteristics and perspectives [26]. Selection of participants for NCD categories was based on self-reporting. Self-reporting of illness aligns with this study’s aim, which explores participants’ lived experiences of illness rather than establishing clinical prevalence [27, 28]. Participants were informed about the study and asked whether they wanted to participate. The participants were identified by co-researchers (community members participating in the research) based on the agreed criteria (S1 Checklist). Those who met the selection criteria were recruited in consultation with co-researchers and interview dates agreed. The recruitment of participants for this study was done from 6^th^ to 10^th^ September 2021, while the three interview sessions were held from 13^th^ September to 14^th^ December 2021.

Participation in the study was based on informed consent. Participants were provided with adequate information to allow them to decide their participation. They were informed about their rights in the study including withdrawal at any time, without reprisals, seeking clarifications or requesting for interviews to be paused if they felt uncomfortable with sensitive topics. Before the start of each interview session, participants were asked to sign a written consent form after being provided with information about the consent procedures. Table 1 below describes the characteristics of participants whose names were pseudonymised to conceal their identity.

**Table 1:** Description of narrative interview participants.

Pseudonyms & Codes	Participant Identities (Disease condition; gender; age; social and income status)
Isata: CBY-STK-F	Isata, a woman in her 40s, suffers from disability related to stroke. She attributed her condition to her physically demanding work as a cleaner. She had since left her job and now relies on the help of her social networks. Her experience with both informal and formal providers have not been very satisfactory, but she prefers services from formal providers, including physiotherapy.
Edward: CBY-STK-M	Edward is in now in his 60s and has lived with comorbidities of disability from stroke and hypertension for several years. He has not worked since he got ill and he is being looked after by one of his older children. He had a late diagnosis of his condition until he had an attack and was hospitalised. He seeks help more often from a drug peddler and a private nurse for different symptoms including pain and cold.
Yeanoh: CBY-HPT-F1	Yeanoh, is a female resident with hypertension. She has not been financially independent since her ill health forced her to abandon her work as a house help and her small business. She faces housing, food and healthcare challenges. She is widowed and is looked after by one of her older children who is also not formally employed.
Margaret: CBY-HPT-F2	Margaret, a young woman lives with hypertensive condition. Her condition was diagnosed after several episodes of ill health and care seeking from informal providers. She faces financial difficulties due to her health condition, coupled with frequent power cuts forcing her to halt her business. She is married but complains that her husband is not supportive in meeting her healthcare needs.
Mbalu: CBY-DBT-F	Mbalu, is in her mid-50s and, has lived with co-morbidities of diabetes and hypertension for about five years. She is widowed and has not been financially independent since she left her engagement as an informal trader. She is currently being taken care of by her grown up children as her health condition had forced her to leave her business.
Abdulai: DZK-STK-M1	Abdulai is retired and lives with disability resulting from stroke (and comorbid hypertension). He was not officially diagnosed but he based his conclusion on the experiences of other people living with similar condition. He lives off a very meagre monthly pension and support from his family.
Salmata: DZK-DBT-F	Salmata, a female in her late 40s has lived with an NCD condition for over a decade. The illness disrupted her business and has now switched to a small business she runs at home. Her late husband also had an NCD condition, which increased her burden of care. Her condition was diagnosed following several incidents of ill health. She visits the hospital occasionally for check-ups and uses bitter herbs and self-medication (use of previous prescription cards to buy medicines).
Thaimu: DZK-STK-M2	Thaimu’s experience with disability related to stroke started long ago when he was young. He retired from his business when his health declined. This has contributed to his current financial difficulty. He visits the hospital occasionally due to financial challenges. His only sources of support are from his family and the little annual rents from his tenants.
Hannah: DZK-STK-F	Hannah retired from her formal employment about five years ago. Her health started to decline after her retirement. The lack of formal diagnosis made it difficult to detect her condition until she fell ill and became immobile. She made a few visits to the hospital but had since stopped due to financial difficulties. Her financial precarity is partly due to bureaucracies to access her pension.
Nancy: DZK-HPT-F	Nancy, who is widowed has lived with the co-morbidities of hypertension and diabetes for over five years. Her condition was diagnosed only after she underwent surgery. The lack of resources has affected her access to formal healthcare services, making her to seek care from informal providers. She has no fixed source of income or support, so she relies on the goodwill of people for food and healthcare.
Mariama: MYB-HPT-F	Mariama’s experience with hypertension started nearly two decades ago when she faced post pregnancy complications. She attributed her condition to financial stress and unmet family needs. She has visited the hospital a few times for check-ups, but most of her healthcare visits are with traditional healers due to her trust in their services. She quit income generating activity due to exposure to criminal activity including robbery, causing her to entirely rely on her family for her upkeep.
Yamba: MYB-STK-M	The co-morbidities of disability from stroke and hypertension have been experienced by Yamba for nearly a decade. He recounted experiences of unexplained attacks and frequent headaches since his teenage years which affected his educational attainment and career ambitions. He managed to find himself minimal wage jobs, despite his health challenges. He interacts more often with “drug peddlers” and less with formal providers.
Sulaiman: MYB-HPT-M	Sulaiman is in his late 40s, and he runs a small business. His long fight with hypertension made him leave his job, which increased his financial stress. The lack of proper diagnosis made him to seek help from informal providers for a long time until his condition was formally diagnosed. Since his diagnosis, he has decided to seek more help from formal healthcare providers, due to perceived long term health benefits.
Ishmail: MYB-DBT-M	Ishmail is in his early 60s and has lived with the comorbidities of diabetes and hypertension for many years. He left a thriving business due to the severity of his health condition. His burden of care had since shifted to his wife who is also ill and struggling financially. He seeks help more often from informal providers and administers home-made traditional remedies to respond to his comorbid condition.
Sarah: MYB-DBT-F	Sarah was a business woman in her hometown until she severely fell ill. The lack of medical facilities back home led her children to make the decision to bring her to the city for treatment. She attributed her hypertensive condition to stress linked to the loss of an inherited property from her father which was sold by her relative who was collecting rents. She concurrently uses traditional and western medicines to deal with her co-morbid conditions.

### Analysis

This study took an intersectional analysis approach informed by the Intersectional Gender Analysis Framework for Infectious Diseases of Poverty Research [3] which provides extensive guidance on how to generate, analyse and interpret findings on disease interactions with poverty, gender and other axes of inequity (S2 Text). A framework approach was applied inductively and deductively to analytically organise and observe patterns in the data and to develop themes [29]. This involved reading and re-reading transcripts to iteratively cluster emerging concepts. A coding framework was initially developed based on the study objectives and insights from the data, which allowed a structure for organising the data and for the emergence of new themes. Prior to this, all interviews were recorded and transcribed. The transcripts were coded in the NVivo 14 software using a coding framework. The analysis made key considerations including gender, disease conditions and age. To operationalise the analysis, each participant was assigned specific case attributes - including community location, age, gender, and disease typology - which served as the primary stratifiers for comparison. These attributes were integrated into a framework matrix, allowing for the systematic cross-tabulation of intersecting identities within rows – as well as against emerging thematic codes within columns. This structured approach facilitated a multi-layered analysis and a rigorous comparison of lived experiences across different demographic subgroups of people living with NCDs. Findings from the themes were then summarised to articulate the linkages and contribution to the study objectives and the adapted conceptual framework.

The novelty of our approach is the adaptation of the Intersectional Gender Analysis Framework for Infectious Diseases of Poverty Research [3] to the study of NCDs in informal settlements in Freetown, to reflect on the complex structural drivers shaping vulnerability to NCDs [15], including poverty, gender and precarious living conditions [13]. The framework was originally adapted from the Intersectionality Wheel [30] to illustrate the intersections of sex identity and gender norms in shaping disease vulnerability (e.g., through poverty, disease exposure and delayed access to care), leading to disease severity, and disability. We built upon the strength of this framework to adapt it to the study of NCD vulnerability and health seeking in informal settlements. The vulnerabilities were layered into macro structural factors (conflict and fragility, urbanisation, and health system barriers) and meso factors (gender norms, poverty, place of living and tenure status) interacting with micro individual circumstances (housing, health status, disability and livelihoods disruptions) to shape the lived experiences of NCDs and health seeking. The adaptation of this framework reflects the need to approach NCD policy from a bio-social perspective, not solely from a biomedical perspective.

### Ethics and safeguarding

The research team discussed during the debrief sessions adhering to ethical procedures and the safeguarding policy of the ARISE consortium by ensuring that research participants and vulnerable adults living with NCD conditions alongside the research team were protected from physical and emotional harm. The research team and participants were informed about referral procedures in the event of safeguarding concerns such emotional harm resulting from participating in the study. The phone contact of the Safeguarding Lead for this study was made available to all participants through the information sheets that were provided. Two of the participants who faced distress during the interviews were given time and space to grieve and were consoled and supported through counselling. Requests were made to refer the participants to professional counselling services. However, no referrals were made as participants reported that they were relieved after discussing their challenges.

Data processing and analysis started from January until August 2022. Data was made available to authors to review and plan the writing of this manuscript. To enhance confidentiality and data protection, adequate safeguards were made to protect the identities of participants in accordance with the data protection procedures of LSTM and SLESRC. This included the deidentification of participants and labelling transcripts with unique identification codes before sharing them with authors for review. The coding framework, and an outline of the manuscript were shared with all authors through email which informed the discussions for authorship. All transcripts and analysed data were stored on Nextcloud, a secure data repository hosted by LSTM.

### Findings

**Fig 1:**
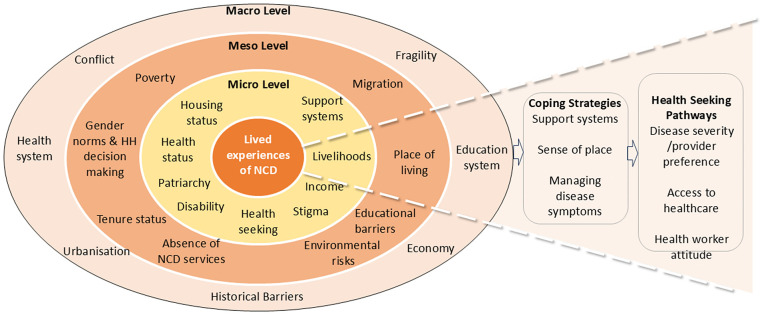
Intersectional Gender Analysis Framework for Research on NCDs (Adapted from WHO’s Intersectional Gender Analysis Framework for Infectious Diseases of Poverty [3].

Findings from this study are presented in three parts. First, we present participants’ lived experiences of NCDs, and how this is influenced, and varies, by gender norms, access to resources and participation in household decision making. Second, we describe participants’ coping strategies and how they are shaped by gender identity, disability, co-morbidities, disease severity, and tenure status. Third, we describe health seeking patterns, influenced by healthcare access barriers and health beliefs. Fig 1 below reflects the key findings presented in the three sections (lived experiences, coping strategies and health seeking patterns), showing the interactions of macro, meso and micro factors. The macro factors represent the structural drivers at city/national level, meso factors at the community level, and the micro factors at individual or household level. Table 2 (at the bottom of the text) also aids the interpretation of the in-text codes. Throughout our findings section, we also draw from S1 Table which further details the health seeking pathways of each of our participants.

**Table 2:** Description of In-text Codes by Alphabetical Order. This table has been provided to ease the interpretation of the codes with the text in alphabetical order.

Code name	Meaning	Code name	Interpretation
CBY	Cockle Bay	M	Male
CC	Community chief	MYB	Moyiba
DBT	Diabetes	NI	Narrative Interviews
DP	Drug peddler	RH	Religious healers
DZK	Dwarzark	STK	Stroke
F	Female	TH	Traditional healer
HPT	Hypertension		

### Lived experiences of NCDs

Lived experiences of NCD reflected the connections between gender norms, structural barriers and their contributions to health outcomes and healthcare decision making. We demonstrate that gender and societal expectations about men and women reinforce power hierarchies in decision making, leading to worsening health outcomes poverty and delayed access to care. Fig 1 above summarises our adaptation of WHO’s Intersectional Gender Analysis Framework, applied in our analysis to explore the relationships between poverty, gender and NCD outcomes, and health seeking strategies (S1 Table). To illustrate these interactions, we present the stories of Isata in Box 1 and Ishmail in Box 2 with the aim of providing a fuller and illustrative understanding of the lived experiences of NCDs.

**Box 1: Isata’s Story**

Isata, a mother of five lives with her husband and children in a rented single bedroom apartment built with corrugated iron sheets or “pan-body”. She lives with stroke related disability, which she relates to mental stress, and precarious working condition. She used to work as a cleaner in a school which involved strenuous activities and exposure to cold due to the prolonged use of water. Although her initial diagnosis pointed to malaria and typhoid, she was convinced that she had stroke by observing symptoms from other affected persons: “*I lost strength in my arms, and my feet became numb, causing restricted movement – and some form of disability…… I observed some of the symptoms from people with stroke. My friend lives with stroke.”*

Isata had a rough childhood because of what she attributed to neglect by her father, and the poor health condition of her mother following her birth, leaving her in the care of her maternal grandaunt. She dropped out from school early and got married at a very young age. The lack of support from her father and the precarious financial situation of her grandaunt deprived her of educational opportunity: *“My father did not care…. I stopped at class five. So, I do not consider it as education”.*

Her relationship with her husband turned sour because of his alcoholic and abusive behaviour, and the neglect of his financial obligations. Isata reflected on her continued stress over the high burden of childcare with little income from her previous menial job, causing her to rely on the support of others. *“I have been the one taking care of my children and myself. One of them is in the University, and the burden is all on me”.*

Isata’s financial uncertainties due to her ill health and the loss of livelihood means limited access to healthcare. The lack of resources has led her to try different healthcare options including traditional healers. Her initial experience with formal healthcare providers was not favourable because of the lack of clear diagnosis, thus increasing her stress about recovery. Despite this, she prefers seeking care from formal healthcare providers whenever she has money because of the relatively better outcomes compared to traditional healers. The outcome from physiotherapy sessions, and a visit from a doctor, she said was helpful. She combined these interventions with spiritual healing and prayer sessions: “W*ith God’s intervention, the pain was fading away, and I experienced a period of relief. Therefore, the combined forces made the difference”.*

**Box 2: Ishmail’s story**

Poverty and migration from rural areas are the common experiences of people living in informal settlements in Freetown. This is reflected in the accounts of Ishmail who left his native village in the north of Sierra Leone to look for a better life. Poverty and the death of his parents cost him the opportunity of formal education. His business adventure was successful, from which he built himself a house.

Soon, Ishmail’s fortune would be hijacked by an uncertain future – ill health. He has lived with diabetes (and comorbid hypertension) for the last 17 years. He explains that his health condition has plunged him into deep financial crisis since he was diagnosed. He relies on his wife and (grown-up children, some of whom are unemployed) for support. Until recently, she has been severely affected by diabetes, almost leading to amputation. Ishmail’s account reflects the shrinking support system for the chronically ill as their carers also face financial crisis: *“My wife is currently not able to do business; we depend on our children, but they too do not have good jobs”.* He feels embarrassed that he is being taken care of by his wife.

His usual experience with hypertension is headache, fatigue, heart palpitation, itchiness and blurry vision. For diabetes, he experiences itchiness when the disease is about to get severe.

Ishmail has had limited interaction with the formal health system, partly because of financial barriers and his health belief. While he believes in the efficacy of western medicine (he goes to the hospital when his symptoms get worse), he also believes in traditional remedies from mango and guava leaves, and bitter roots for the treatment of diabetes and hypertension: “I am not saying medicines from the formal health service providers are not good…. *but the herbs are effective for diabetes and hypertension, and I always see great improvement”.*

### The interplay between gender and poverty: barriers to education

Health outcomes and the capacity to respond to health crises are often similar for both men and women. However, gendered hierarchies and the influence of other factors such as minimal education often meant that the route to this outcome was different for men and women. Throughout our findings, women expressed difficulties in accessing educational resources, as in the case of Isata (Box 1), which limited their capacity to respond to health crisis. For example, women living with co-morbid diabetes and hypertension were particularly concerned about their exclusion from formal education because of the long-term impacts of patriarchal norms, beyond their healthcare needs, affecting their upward social mobility and the ability to respond to future health shocks.

Older women with co-morbidities of diabetes and hypertension, were vocal about family and patriarchal notions of women and the institution of marriage which encouraged arranged marriages for them when they were very young, with economic stability often described as a key driver. This was the case with Sarah who lives with the comorbidities of diabetes and hypertension:

*“I got married to a man who already had two wives; I was the third. He was the same man who took me from school. I am the last child of my mother and when she died there was no money to continue my education, so I was forced to marry this man.” (NI_MYB_DBT_F)*

Caregiving responsibilities were also reported to have unduly affected women’s educational advancement, which also impacted their future career prospects. One woman living with diabetes (and comorbid hypertension) *(NI_CBY_DBT_F)* noted that her role as the prime caregiver of the child of her deceased elder sister took a toll on her schooling, eventually leading to her withdrawal from school.

Men across NCD conditions also shared their experiences about the challenges of accessing formal education. However, these experiences seemed to be tied largely to cycles of poverty, amplified by personal health circumstances, rather than driven solely by patriarchal disadvantage as in Ishmail’s story (Box 2). Patriarchy for some men was described in terms of taking on family responsibility at an early age, disrupting educational and career prospects, particularly at the loss of a breadwinner during childhood:

*“My parents died, and I was left with my uncles and aunts who also had their own families to take care of. I didn’t want to overburden them, so I came to Freetown. Thankfully, I learned driving skill from which I supported my family back home.” (NI_DZK_HPT_M)*

Men also described personal health conditions as factors acting as barriers to educational access and completion. Many adults in Sierra Leone engage in schooling to attain basic literacy, in response to lost opportunities to be educated during their formative years. An elderly man with disability from stroke (and comorbid hypertension), noted that he faced strange health problems while in school, climaxing to several attacks of unexplained illness and headaches forcing him to leave school early:

*“I left school because of this illness. I think I had the first attack when I was preparing to write the ‘Ordinary Level’ examinations, but the agonising headaches forced me to discontinue the preparations for the exams.”* (NI_MYB_STK_M).

### Financial hardship: The interplay between gender, poverty, ill-health and fragility

Experience of financial hardship was common for participants, and this was due to both non-health and health factors. First, as an outcome of social and political instability such as the civil conflict, forced migration and the lack of livelihood opportunities. Second, as an outcome of chronic long term health problems, often resulting in complex health seeking journeys, with ongoing oscillation between provider types as emphasised in S1 Table.

Participants stated that the civil, conflict forced migration, and the loss of livelihood opportunities severely impacted their financial status. Participants migrating to urban settings expressed that they had high hopes of better living conditions, but their hopes were shattered due to the new layers of vulnerability faced in urban informal settlements, including poor housing conditions and access, and insecure livelihoods. For some participants affected by NCDs, living in an informal settlement brought about new challenges which contributed to anxiety, and stress. Men and women in these conditions spoke about difficulties faced to secure a decent living due to livelihood disruptions. However, a few men were lucky enough to establish and sustain small businesses, whilst others felt less fortunate, undertaking menial jobs such as night security, laundry, and other forms of domestic labour as an immediate solution but with ongoing financial insecurity. For example, older generations of men who migrated to Freetown before the war, spoke about the long-term impacts of the poverty-illness nexus related to insecurity, including the war and military coups, leaving them unemployed and susceptible to ill health. These experiences were described by two men living with disabilities related to stroke and hypertension:

*“I became jobless since the military coup in 1992, and I have not been able to get a job ever since. To survive and sustain my family, I engaged in menial jobs like ironing, laundry, and other domestic chores.” (NI_CBY_STK_M_02)*

*“We suffered a lot during the war. After I lost my job at the start of the war, I decided to go into a small business, but the rebels destroyed everything when they attacked our town. I was left with nothing. When I came to Freetown, life was difficult because I knew no one and I had no job which got me frustrated.” (NI_MYB_HPT_M)*

Women’s accounts of poverty and financial difficulties were centred on historical experiences, such as conflict and caregiving. Food and housing difficulties, and personal circumstances such as widowhood, and single parenting also drove financial instability. Regardless of whether women had been born in Freetown or migrated to informal settlements later in life, they experienced similar financial difficulties which they believed were negatively impacting their quality of life.

Some women were engaged in precarious livelihood activities due to financial challenges, which exposed them to new layers of vulnerability, as described by Mariama who lives with hypertension *(NI_MYB_HPT_F):*

*“I live with my husband who is jobless; this difficult situation left me with no choice but to go into wood harvesting to sell and survive with my family…...I left wood selling due to fear of kidnappers in the bush. I also engaged in stone quarrying for four years until when I gave birth to twins and later developed health complications.” (NI_MYB_HPT_F)*

The absence of family to support with childcare by women who had become widowed during the war and migrated due to the conflict led to gendered financial and housing challenges.

*“When we came to Freetown, it was extremely difficult for us. I had engaged in buying “Mina fish” (small sized and low cost) at the wharf and hawked them across several neighbourhoods to make a living. I discontinued fish hawking and started selling cake which I continued till we moved into this community.” (NI_CBY_DBT_F)*

Poverty and financial instability were often exacerbated by NCDs, further disrupting livelihoods and pushing people further into the poverty-illness nexus. Women and men stated that because of their recurring health problems, there were increased financial pressures due to livelihood insecurity, and the additional costs of health seeking (discussed later, and in S1 Table). Despite these shared financial difficulties for both men and women, outcomes were different related to the severity of health problem, alternate income and support systems. Salmata, (*NI_DZK_DBT_F)*, a woman living with diabetes (whose husband was also diabetic) narrated her experience with ill health which had caused her to abandon her food retail business in the market:

*“I quit selling rice at the market because of diabetes. My health condition was seriously affecting my ability to do business.” (NI_DZK_DBT_F)*

Experiences of chronic long-term ill health and their impact on livelihood outcomes were described by some women as causing them to lose all hopes of social mobility and a better life, as described by Isata:

*“Honestly, I have no plans because there is nothing, I can do to generate income. I have been in this condition for over a year now and my health is not improving……..” (NI2_CBY_STK_F)*

Within gendered categories, differences in experience were also evident. For example, men who were low-income earners and with high numbers of dependents were more likely to be impacted by financial hardship than men who were taken care of by their families. For Sulaiman, who lives with hypertension, *(NI_MYB_HPT_M),* having to leave his job due to poor health condition meant huge financial consequences (S1 Table):

*“The reason why I resigned from my job was because I could no longer work and seek medication at the same time. This is my fourth year since I became ill and during this period, it has been difficult to find a living.” (NI_MYB_HPT_M).*

### Gender norms and intra-household decision making

Gender was identified as an underpinning power dynamic within households, shaping access to and control of resources, and response to disease conditions. Findings here reflect these dynamics, as they relate to masculinity, conflict and changing household dynamics, and decision making.

Masculinity and gender dynamics were viewed as important in the way households shared responsibilities. Across settlements, stereotypical gender roles were evident, with women expected to nurture children, and men serving as breadwinners. Masculinity was often perceived in terms of men’s physical and financial powers. Thus, when men became unemployed or financially vulnerable due to long term ill health, their masculinity was challenged as this meant a diminished role within the home. Consequently, many men felt pressured to keep up with these economic obligations, which often caused marital conflicts, including denial of sex from their spouse, further challenging masculinity. One woman living with hypertension remarked that because gender norms bring expectations on women to always prepare for sex with their partners, failure to conform can result to conflict. In her case, sexual agency tended to be used as a strategic resource to seek her husband’s support towards her healthcare and financial needs:

*“Sometimes, I tell him that he is not doing the right thing, because when you are in a relationship, it is your responsibility to care for your partner, especially regarding health issues. He often talks about extended family burden, but I always tell him that I am his immediate family. So sometimes I deny him sex which often results in conflict.” (NI_CBY_HPT_F_02)*

Some women ascribed their increased financial burden to ill-health, particularly when their partner was not working hard enough to meet household needs. For example, Isata (Box 1) who had experienced a disability resulting from stroke attributed her condition to her precarious working conditions, combined with her husband’s lack of support:

*“My husband does not work hard to care for me and our children. Hence, I had to engage in menial jobs. That is what I have been doing to make ends meet which may have resulted in this illness.” (NI_CBY_STK_F).*

The intersections of masculinity, perceived loss of power, ill health and financial vulnerability were crucial to how men viewed themselves. Therefore, the negotiation of power between men and women was often difficult, particularly when men felt vulnerable to ill health, impacting how they cope with ill health. Moreover, men sometimes found it troubling to be looked after by their wives when they faced health crisis, signalling a “loss of power” and change in their social status. This was described by Salmata *(NI_DZK_DBT_F*), whose husband construed her as wanting to ‘control him’ because she was making decisions when he was severely ill with diabetes:

*“He thinks that I want to be in control of the home because I provide financial contribution. but that is not the case…. He always becomes angry; He tells me sometimes that I feel I am the man of the house…. Because my financial contribution to the home is more than his, he always feels insecure.” (NI_DZK_DBT_F*)

### Living with disability

NCD related impairments (or disability) were described in terms of major deviations from social and physical functioning, notably the disruptions in mobility and livelihood activities (as described above). These disruptions - sometimes leading to disability, were often described as sudden episodes of physical impairment attributed to shocks from collapse, which were associated with heart attacks and stroke. These conditions were often diagnosed late due to healthcare access barriers (S1 Table). Different accounts from women, particularly those with disability from stroke referred to disability as “life changing events” that often plunged them into long periods of reduced physical and social functioning:

*“It was just one night, when I was sleeping. I woke up with a sudden attack and fell on the ground. Immediately, my hands and feet became numb, and I couldn’t move. I couldn’t do anything. This sickness has really changed my life.” (NI_DZK-STK-F)*

Similar accounts of shocks related to disability were expressed by men particularly, those living with hypertension and disability from stroke*:*

*“I collapsed while using the rest room. I had a very sharp pain like an electric shock at the back of my neck” (NI_MYB_STK_M2)*

Women and men particularly those living with disability with stroke also reflected on the gradual progression of pain, which advanced into physical disability, including the loss of mobility, and eye impairment. The rugged nature of informal settlements, coupled with material and social deprivations (e.g., the lack of assistive technologies) for people with diminishing health were shown as factors that intersected to shape constrained access to healthcare. As described by Isata who lives with disability related to stroke (Box 1), these factors negatively impact health seeking and worsen health outcomes.

*“Both of my legs are weak, and I cannot stand without holding or leaning on something. Before this time, I was able to walk alone even though at a slow pace but right now, I can only do that by holding a stick or getting someone to hold me.” (NI2_CBY_STK_F)*

Beyond the physical impairments, disability from NCDs was viewed as a new form of identity that induced fear and the feeling of stigma. This description was common with women living with diabetes and disability from stroke, which they believed were physically draining, and contributing to a change of their physical outlooks. For women living with diabetes, the feeling of looking pale or emaciated frequently caused a sense of shame and stigma, as described by Salmata: *“My body was rounded, but it has been depreciating since I had diabetes (NI_DZK_DBT_F).*

### Coping strategies

Findings on NCD outcomes and coping strategies were linked to the Human Model of Disability which frames physical experiences of disease conditions as impairment, particularly if they are affected persons due to poverty, stigma and environmental conditions (S1 Table). We have added another dimension to this framing by considering factors such as individual identities, (including poverty and physical manifestations of disease), which in turn shape support systems, sense of place/belonging and knowledge about disease management as factors shaping the ability to cope with NCDs (See Fig 1)

### Support systems “My wife who has been helping me has been sick too”

The nexus between NCD-poverty experiences and weak support systems were evident, as participants described their experiences of struggles and desperation for help. Support systems were linked to the nature of help from family and friends during health shocks. Health shocks were described in relation to major physical or health disruptions impacting the ability to seek livelihood and cope with disease. Women with diabetes, hypertension and disability from stroke spoke about being stretched to the limit due to limited or depleted support from family and well-wishers. Diminishing support was attributed to the demise of a close family member, financial difficulties by their benefactors, or the reluctance of family members to help.

*“I was having money from people who were coming to visit me; they helped me financially and with food and medicine, but now they don’t come anymore.” (NI_DZK-HPT-F).*

Where women felt less supported stress was often the case. Some women described having to survive with almost nothing. Women who were widowed and childless were more likely to be worried about diminishing support.

Men with disability from stroke, hypertension and diabetes also described coping strategies towards disease response. Support for men included generous financial contributions from well-wishers, including people with longstanding relationships and family members. One man with disability from stroke noted that donations from their benefactors often went towards buying food and medicine, and sometimes paying rents:

*“When I was well, I was good at ironing, so I used to help a man to iron his clothes. He has been so good to me, and he is the one who has been paying my house rent.” (NI_CBY_STK_M_01).*

While men expressed being in dire need, many felt discouraged about being looked after by others. This was particularly the case when they realised that caregivers themselves struggled to meet their own healthcare needs. The nature and length of support received for men depended on the health and financial status of their caregivers, some of whom were often stressed about their own health, and people in their care, as in the case of Ishmail (Box 2). Despite these challenges, caregivers went beyond their limit to support their loved ones with long-term health problems:

*“My wife and children have been paying my bills since I no longer do business to earn money. So, now I rely on them for financial support. However, my wife who has been helping me has been sick too, with diabetes, chronic cold and eye problem. She even underwent an eye surgery.” (NI_MYB_DBT_M)*

### Sense of place/tenure status

Insecure tenure was reported as a challenge shaping the experiences of women and men living with NCDs in informal settlements. Living in one’s own house was tied to the feeling of respect within their community. A sense of belonging to a place was enacted through secure housing tenure, which also shaped how people coped with long-term illnesses. Women’s experiences of housing difficulties reflected the histories of war, forced migration and widowhood, causing frequent relocations. Women who were widowed or childless were more likely to experience housing challenges because of the intersections of ill health, loss of livelihoods due to disease intensification and the lack of support:

*“We have no permanent place to live, which is why we move from one pan-body house (corrugated iron sheet) to another. We lived in several places in Freetown, before we finally moved into this community. The person who rented this place said the land was not his. So, we may be asked to leave at any time the land is requested.” (NI_CBY_DBT_F).*

The precarity of housing, influenced by gendered hierarchies triggered a sense of helplessness, leading to stress and discomfort, as expressed by one woman, who is widowed and living with hypertension:

*‘‘I spoke to the landlord about renovating the single room I was living in as part of my rent, and I was given permission. Upon completion, I presented my receipts to the landlord to agree on my new tenancy. To my surprise, I was asked to start paying rent at the same time I finished the renovation. The new landlords (after the death of their mother) issued eviction letters to all tenants in the compound’’ (NI_CBY_HPT_F_01)*

Men’s accounts showed that owning a house ignited a sense of comfort and belonging. However, men with insecure housing tenure, and with limited support lamented about increased challenges, and coping with ill health more difficult. While men with secure housing tenure experienced health shock, such as stroke, they were encouraged to cope due to the feeling of respect, due to secure tenure and familial support:

“*I think the main reason why people respect me in this community is because of my house. If I had not built this house, with my condition now, I would have returned to my village. Had it not been for that, my situation would have been much worse.” (NI2_DZK_STK_M2)*.

### Managing disease symptoms

During health shocks, internalisation of disease symptoms were key in coping with and managing the physical manifestations of ill health. For many women across all NCD conditions, headaches, body pain, dizziness, fatigue, spinning head and blurry vision were the common symptoms that informed them about the intensity, which often triggered health seeking (S1 Table). Women with diabetes often spoke about “spinning head,” while those with stroke identified severe body pain. Isata who lives with disability from stroke identified her usual symptoms of disease intensity as pelvic pain and knee cramps:

*‘‘What affects me more is pelvic pain; it hurts me, and I also experience cramps in my knees’’ (NI_CBY_STK_F)*

Response strategies included self-care strategies including light physical exercises, such as walking, medication and adequate rest. Some women, particularly those with hypertension and stroke spoke about relying on painkillers such as paracetamol to deal with recurring headaches and body pain (see self-care practices using medicines from the pharmacy, S1 Table) and the use of towel in warm water to gain relief from numbed fingers, believed to be related to severe cold:

*“Headache is the main symptom I face, but it usually lessens as soon as I take paracetamol. I also feel severe pain on both of my feet for which I use painkillers.” (NI_CBY_HPT_F_01)*

*I cannot fold my fingers unless they are soaked with warm towel which was very painful (NI_CBY_STK_F)*

For women with diabetes, care seeking from formal providers was somewhat consistent due to the need to be constantly abreast with their health status. Response to diabetes also related to observing their blood sugar, particularly when they noticed symptoms such as spinning head or fatigue. Those who had blood pressure machines used these observations to check their blood sugar with the help of a private nurse at home (as described by Salmata in S1 Table).

Men’s symptoms of disease intensity were similar to those of women, and notably included difficulty in walking, headaches, increased heart palpitations, numbness in the arms and legs and generalised body pains. Care seeking for men with disability from stroke was more from drug peddlers, and nurses at home to treat pain, heart palpitations and numbness. Seeking care from formal healthcare providers was therefore informed by disease intensity or the obtainment of health advice, as in the case of Ishmail (S1 Table). Similar experiences of managing symptoms such as pain were summarised by Sulaiman (S1 Table) who had severe difficulties and uncertainties with the diagnosis and treatment of hypertension:

*‘‘I can’t even walk for a long distance, and if I attempt to do so, my whole body becomes numb, and my feet suddenly begin to shake and become numb as if they have no life’’. (NI_MYB_HPT_M).*

Men also described using painkillers and menthol-based gels, referred to as ‘hot rubs’ to deal with generalised body pains, believed to be related to NCDs. Understandings about disease intensity helped men affected by NCDs in not only shaping response to specific symptoms, but in coping with changing weather patterns, as summarised by a man living with disability related to stroke.

*“I have a hot rub which I apply on the affected part of my body daily. I also buy pain killers from the pharmacy. I feel more pain during the dry season because the intensity of the sun always causes headaches and palpitation of my heart.” (NI_DZK_STK_M1)*

Some men including those with hypertension and diabetes also described the use of light exercises such as walking to deal with body pain. Ishmail who lives with diabetes (and comorbid hypertension) cited the use of self-made powdered herb tea as helpful in urinating easily to reduce his high blood sugar and “feel normal” (S1 Table)*.* Since NCD conditions are long-term illnesses, designing effective ways to live with them is vital. In this section, patients designed different strategies to observe and manage their disease symptoms. In the next section, we shall see how these understandings shape care seeking pathways from multiple providers (formal and informal).

### Health seeking pathways

In this section, our findings show that syncretic seeking practices were observed among women and men (S1 Table). Syncretic practices involved the concurrent use of multiple treatment regimens, including traditional, religious, and biomedical services, with the hope of attaining satisfactory outcomes. These seeking practices were influenced by interrelated factors, (as shown in Fig 1 and further elucidated in S1 Table). These seeking pathways were influenced by the following: 1) Disease severity and provider preference 2) Barriers to healthcare (influenced by financial limitations, leading to delayed diagnosis and treatment), and 3) Health worker attitude.

### Disease severity/provider preference

Across participant groups, including gender, diverse seeking practices were observed for the treatment of diabetes, hypertension, and disability resulting from stroke. Participants’ disease conditions and severity, coupled with preference of certain providers influenced how health seeking decisions were made. For women with diabetes (and comorbid hypertension), treatment from private nurses at home, and self-care practices, using old prescription cards to buy medicines from pharmacy was observed (as in the case of Salmata in S1 Table). However, they kept a close contact with their formal providers to check their blood sugar. Perceptions of relatively better outcomes for the treatment of diabetes, including proper diagnosis and prescription of medications influenced care seeking from formal providers by women with diabetes. This experience was described by one woman with diabetes (with comorbid hypertension):

*“Since I started taking the medicine prescribed from the doctor, I have been feeling better, and I always take it as prescribed” (NI_CBY_DBT_F).*

However, when women with diabetes sometimes experienced side effects, (e.g., excessive weight loss), slow recovery or recurrent symptoms during biomedical treatment, they were likely to seek alternative care such as traditional remedies, including bitter roots soaked in water, believed to be helpful in reducing their blood sugar, as shown by Salmata in S1 Table. For example, Salmata was concerned about side effects resulting from repeated use of diabetic medications leading to discomfort:

*“I have taken the medicines for diabetes for a long time, but my body is still deteriorating. I spoke to the doctor, and he advised me to start taking vitamins.” (NI_DZK_DBT_F)*

Self-care practices using medicines from the pharmacy were also identified as critical part of therapy management, due to financial barriers, unsatisfactory outcomes and the involvement of close social networks in therapy management, as shown by Nancy (NI_DZK-HPT-F) in the treatment of hypertension (S1 Table)*.*

Women with hypertension also practiced syncretic seeking in response to specific symptoms such as heart palpitations, headaches and body pain. While care seeking from formal providers was recognised as important in ensuring overall health benefits, treatment avoidance was observed by some women as a strategy to enact self-ignorance about their illness status, to avoid the consequences of high financial costs associated with long-term illness, as observed in the case of Mbalu (NI_CBY_HPT_F1) (S1 Table).

*‘‘I was taken to a hospital where they examined and referred me to another hospital, but I refused to go there because I was afraid of being admitted, as one of my children was diagnosed with typhoid and malaria which nearly resulted in madness. So, I feared being diagnosed with a severe illness that could have gotten me admitted’. (NI_CBY_HPT_02).*

The choice of familiar providers such as drug peddlers or private nurses was believed to bring comfort. For example, drug peddlers were selected to treat pain and headaches; herb sellers and pharmacies to obtain medications to manage high blood pressure through self-care, while hospitals were the final point of contact for the management of disease severity, as illustrated in S1 Table. Self-care, using herbal remedies, including ginger, garlic and honey were thought to be useful in providing relief from frequent heart palpitation, reducing body weight, while bitter roots (e.g., “gbangba”) were believed to be effective in managing high blood pressure, and in “cleansing the system” (NI_CBY_HPT_F_02). This practice was observed by some women with diabetes and hypertension as described in S1 Table.

Women living with disability from stroke, however, interacted with physiotherapy services to improve mobility, following shocks such as collapse. Immediate outcomes were reported to be good, although patients were concerned about slow recovery, as described by Isata and Hannah, and further illustrated in S1 Table:

*‘‘What I have observed is my ability to raise my legs and take a few steps as this was an impossible thing for me to do some time ago. However, I have not been feeling well for the past two days due to severe cold, and abnormal heart palpitation. I feel so sick’. (NI2_CBY_STK_F)*

Drug peddlers, however treated women for pain and cold, using painkillers and hot rubs. These remedies were sometimes combined with prayers and healing sessions by women with disability with stroke (see S1 Table), particularly when recovery from traditional and formal healthcare providers was observed to be slow.

Men across NCD categories appeared to have limited interactions with formal healthcare providers, as shown in S1 Table. For men living with disability related to stroke, the temporality of interactions with formal healthcare system was evident, based on financial limitations, health beliefs and proximity with alternative care systems. Care seeking was more from drug peddlers, and treatment from private nurses at home, as shown by Abdulai’s story (S1 Table). Frequent care from drug peddlers was reported due to their perceived experience and empathy in dealing with patients:

*“I buy drugs from a drug peddler who sells medicines to the community residents. His is very experienced, and his drugs are very effective. He once offered me a card of drugs (10 tablets) when I did not have money.” (NI2_CBY_STK_M).*

While men with diabetes, disability related to stroke (and comorbid hypertension) interacted with formal providers in response to different episodes of disease intensity, they showed strong agency towards improving their interactions with alternative providers (e.g., drug peddlers, herb sellers and private nurses providing care at home (unauthorised treatment to patients outside of the formal clinical setting) to improve their response to their daily health problems, as illustrated by Abdulai in S1 Table. For Ishmail, who lives with diabetes (and comorbid hypertension), knowledge of therapy management using traditional remedies (e.g., guava, moringa and mango leaves) was vital in enhancing his illness and treatment journeys:

*“When my feet get swollen, I drink this medicine, and the swelling reduces quickly. It makes me feel better after urinating freely. With the help of ALLAH, my condition is getting better. I don’t remember going to the hospital or pharmacy to buy drugs very often because I trust the traditional medicines that I use.” (NI2_MYB_DBT_M)*

Similar approach was observed with Edward and Abdulai (S1 Table) who have created beneficial relationships with nurses and drug peddlers to enhance timely access to care, and critical health advice. However, one participant who identified with hypertension (Sulaiman) provides an interesting illustration of shifting health beliefs. He initially had a firm belief in traditional healers and drug peddlers, but this was radically shifted towards the formal care system, due to treatment failure. His decision to stick with formal providers was based on cumulative and long-term health benefits (despite the initial uncertainties about diagnosis and high costs) as described in S1 Table.

### Healthcare access

Healthcare access, influenced by distance, healthcare availability, financial barriers, and geographic impediments was vital in shaping health seeking pathways. Women and men across NCD conditions were affected by these factors. However, high costs of healthcare were frequently mentioned as part of the major limitations, as described in S1 Table. Women with diabetes, and hypertension expressed frustration about missing appointments with their doctors for routine checkups, and physiotherapy sessions by women with disability resulting from stroke due to high costs (S1 Table):

*‘‘I was asked to make regular visits to the hospital to monitor my blood pressure, but it has taken a while since I visited due to the lack of money’’. (NI_CBY_HPT_F_02).*

Similar constraints were expressed by men across all disease conditions, particularly those with disability from stroke, whose financial barriers to healthcare were coupled with mobility challenges due to geographic impediments, as noted by Abdulai in S1 Table:

*“Difficulty to walk prevents me from visiting the hospital, which is why I don’t go there often” (NI_DZK_STK_M1)*

Therefore, participants described diverse strategies to access healthcare, including treatment from formal and informal providers. Self-care practices emerged as an important seeking strategy, through the purchase of medicines from pharmacies and herb sellers. Self-care involved the replication of treatment using previously administered treatment by a healthcare provider. This practice was observed notably among women with different NCD conditions to treat specific NCD and non-NCD symptoms as described by Salmata who lives with diabetes (S1 Table):

*“I have already known the medicines to take for diabetes. I usually buy Metformin and Diamine tablets at the pharmacy. Through my previous visits to the hospital, I am now familiar with the dosage. When I don’t have money to go to the hospital, I use the prescription to buy the medicines.” (NI2_DZK_DBT_F).*

Concurrent seeking from nurses and drug peddlers by men living with disability from stroke was evident, believing that treatment procedures and outcomes from these providers were the same as those provided in the hospital. This was described by Abdulai, and further illustrated in S1 Table:

*“I do not have medicines specifically for the stroke, but I have pain killers which I buy from drug peddlers living in this community. I also have a trained nurse in my neighbourhood who gives me injection” (NI_CBY_STK_M_02)*

Across all participant categories, the lack of collaboration among providers (e.g., formal and informal) and referral pathways were factors further complicating health-seeking journeys, by disrupting the continuum of care, and increasing the likelihood of catastrophic spending, and overall negative patient outcomes. Limited access to formal healthcare, influenced by perceptions of high costs of care and service inefficiency, contributed to late diagnosis, leading to disease intensification and chronicity, particularly for women with disability from stroke (See S1 Table.

### Health worker attitude

The attitude of some formal health workers towards patients including the lack of respect was reported as a factor that turned them off - making them to switch to alternative providers, including drug peddlers, purchase of medicines from pharmacies for self-care, and treatment from private nurses. For women who experienced negative attitudes from health workers, this sometimes led to treatment avoidance, leading them to buy medicines from the pharmacy for self-care. Women were concerned that health workers prioritised money over their healthcare needs, as noted by one woman with hypertension:

*“When you go to the hospital, the first thing the doctors will tell you is to be admitted because of the money. The cost of admission, including fees for bed are very high. Hence, I go to the pharmacy to buy medicines.” (NI2_CBY_HPT_F1)*

Experiences about health worker attitude were also expressed in terms of lack of interest and empathy by nurses and doctors. An elderly man living with disability from stroke recounted his experience during admission at a hospital in Freetown:

*“The nurses lack empathy. They often watch movies on their laptops while patients suffer. They lack patientcare understanding. I was there when a patient in the same ward passed away; no nurse came to his aid to provide him the needed support.” (NI_CBY_STK_M_01)*

However, some women seeking care from formal healthcare providers, particularly at private clinics reported overall satisfaction with how they were treated by health workers. Sentiments about satisfaction with health worker attitude related to what they translated as “good treatment” due to the perceived politeness and friendliness of some healthcare workers:

*“I feel good about everything at the hospital because the doctor is so polite.” (NI2_CBY_DBT_F).*

Similar experiences of satisfactory health worker attitude was expressed by a few men living diabetes and hypertension. These included the provision of quality drugs, and occasional treatment on credit basis:

*“The doctors have been very kind to me whenever I visit the hospital. They always provide quality drugs and sometimes give me medicines on credit, and I pay back later when I have money.” (NI_MYB_DBT_M)*

## Discussion

This study was undertaken to understand the lived experiences of people affected by NCDs in informal settlements in Freetown. We applied an intersectional analysis to our exploration to consider how disease experience is shaped by intersecting axes of identity (gender, poverty, disability, and place) and amplified by patriarchal disadvantage and livelihood shocks, causing different health outcomes and response capability by men and women. We adapted the Intersectional Gender Analysis Framework for Infectious Diseases of Poverty Research [3] to understand the nexus between NCDs, gender and poverty. The adapted framework provides valuable insights into the theoretical and practical approaches for integrating gender and intersectional analysis into chronic disease research. The framework highlights the complex nature of NCDs, and how they shape vulnerabilities, and health seeking.

While efforts are being made within LMICs to address gendered social disadvantages such as economic barriers, these considerations are often siloed and do not consider the interactions between financial barriers influencing and amplifying NCD outcomes [31]. Within our study, the application of the Intersectional Gender Analysis Framework enabled us to explore the complex social and structural drivers of ill-health, underpinned by gendered barriers in Sierra Leone. As health and social inequities continually impact negatively on marginalised urban populations in LMICs, an intersectional analysis of poverty, gender and NCD relationships is imperative [7, 14]. Studies have applied these approaches across contexts to understand the different layers of disease vulnerability by examining the connections between Neglected Tropical Diseases (NTDs), disability and mental health [32]; gendered exposures to infectious diseases and outcomes [33]; stigma related to co-morbidities of obesity, diabetes and hypertension [34]; and HIV, influenced by poverty, gender identities, and disability [35, 36].

Insights from the findings above align with ours, as they show strong linkages between poverty, gender stereotypes and the aggravation of NCD disease burden, which influence health seeking strategies. The poverty-illness nexus was shown through a reduced capacity to respond to health crisis by women and men living with various NCD health problems [37]. NCD vulnerabilities in informal settlements indicate entrenched inequities experienced through healthcare access barriers, housing difficulties, and livelihood shocks, which limit timely and adequate access to healthcare. NCD health problems therefore amplified these pre-existing crises.

Women’s lived experiences of NCDs reflect historical and patriarchal disadvantages through their limited financial autonomy, barriers to healthcare decision making and treatment access, compounded by gendered impacts of conflict and migration. Men’s experiences reflected an erosion of social status and financial autonomy, which are both highly gendered, resulting in limits to healthcare access and coping. Our findings are supported by an earlier study that suggests that gendered barriers, including minimal education, lack of financial autonomy, and limited support systems contribute to different capabilities by men and women to respond to ill health [22].

Gender differences, influenced by masculinity were also key in shaping household power dynamics and healthcare decision making. Power dynamics - reflected in the division of roles between men and women - and historical barriers to accessing resources were key factors shaping women’s healthcare decision making [38]. For men living with NCDs, including disability related to stroke and diabetes, the intersections of masculinity, perceived loss of power, ill health and financial vulnerability were crucial to how they viewed themselves. Since men played dominant roles in household decision making, financial collapse and failing health signalled “loss of power” through diminished decision-making power. This was often a source of marital conflict, impacting men’s ability to cope with NCD health problems.

Consequently, coping with the burden of NCD conditions in informal settlements is undermined by financial barriers, limited support systems, and the lack of NCD services which increased uncertainties about treatment access in informal settlements. Healthcare access barriers faced by people living with NCD, combined with daily challenges (e.g., poverty, livelihood shocks, and housing insecurity) limit physical and social functioning, often leading to disability. The Human Development Model of Disability [39] describes disability resulting from health conditions in terms of deprivation of support in enhancing capability and functioning. The model of disability aligns with our findings, as people living with NCDs sometimes felt disabled due to deprivation in meeting their social and healthcare needs. Therefore, participants often adapted different strategies to cope with the burden of NCDs, including care seeking from multiple providers to address symptomatic conditions of ill health.

Syncretic seeking practices were observed among women due to severity of illness, financial barriers, and uncertainties about diagnosis leading to unsatisfactory treatment outcomes. For example, women living with disability from stroke, interacted with diverse providers, including drug peddlers, traditional healers, religious healers and physiotherapy services, to treat symptomatic conditions such as cold, pain, headaches, and mobility challenges. Similar syncretic seeking pathways were observed among men across all NCD conditions. While some men had relatively better advantage over women in terms of secure housing tenure, social networks and familial support in coping with the burden of NCD, care seeking was more common from drug peddlers, and private nurses providing care at home, particularly those living with disability related to stroke. Care seeking from formal healthcare providers was therefore informed by crisis, and the obtainment of vital health information which helped in the Internalisation of self-care. These findings therefore indicate that health systems need to understand the complex drivers shaping NCD vulnerabilities and health seeking practices, to help in designing context specific healthcare interventions.

Such adaptations are critical in addressing structural barriers to healthcare, such as gender differences and minimal access to healthcare. For example, masculinity, with respect to health has often tended to focus on power dynamics and the social disadvantages faced by women, without also exploring how it negatively affects men [38]. Therefore, achieving global health policies and interventions that prioritise gender equity to improve population health outcomes remains a challenge [38]. Policies with limited focus on gender and masculinity have the potential to limit men’s access to healthcare. Chikovore et al., [40] cited stigma and the feeling of not being welcomed as factors that inhibit men from seeking care from primary healthcare facilities for conditions such as tuberculosis (TB) and HIV - as these facilities are believed to prioritise maternal and child health issues. Similarly, worse health outcomes have been identified among men living with NCD conditions because of limited or delayed access to healthcare [41]. Similar patterns were observed in this study as men with hypertension, diabetes and disability resulting from stroke were more inclined to interact with alternative providers such as drug peddlers, private nurses, and traditional remedies due to barriers to formal healthcare services, shaped by financial challenges, distance and geographical impediments.

The strength of this study is the application of a gender and intersectional lens to the study of NCDs in urban informal settlements. The application of the Gender and Intersectional Analysis of Infectious Disease of Poverty Research [3] in a new context (i.e., in informal settlements in Freetown) has enhanced our understanding of the disease-poverty nexus. An important contribution is the application of this framework specifically to NCDs, which brings a new understanding of NCD interactions with poverty and gender inequities.

By building on the framework’s previous application to infectious diseases, its adaptation to NCDs in Freetown has provided additional insights into how structural inequities worsen disease outcomes, particularly for women. One of the leanings drawn from this study is that NCDs impact women and men differently which means that the delivery of healthcare services must be designed in a people centred approach that integrates the perspectives of patients and providers for an effective management of long-term illness (United Nations, 2015). Finally, health systems must be gender responsive to ensure that the healthcare needs of patients with diverse social identities are adequately addressed.

One of the limitations of this study is its limited geographic scope - being conducted in urban informal settlements - which excludes the diverse experiences of other urban populations living with NCDs in Freetown. However, conducting this study in urban informal settlements helps to address evidence gaps on NCDs in complex and marginalised urban contexts. Future studies should explore the voices of urban residents across varying layers of vulnerability to understand how these vulnerabilities are shaped.

## Conclusion

Findings from this study reveal strong linkages between poverty, gender inequality and disability in shaping the burden of NCDs. Gender norms and patriarchal disadvantages reinforce power hierarchies and exacerbate poverty and negative health outcomes. Health systems should adapt to the challenges that reinforce the burden of NCDs in rapidly changing urban environments (e.g., poverty and social inequalities) by ensuring that healthcare delivery meets the needs of patients with diverse social identities. This aligns with our findings which reveal that women’s lived experiences of NCDs reflected gendered historical disadvantages (e.g., patriarchal oppression) that limit financial autonomy and healthcare access by women, while men’s experiences were influenced by changes in social status, due to conflict and migration, and financial instability, limiting their access to healthcare. This requires that health systems should design gender responsive strategies to address NCD burdens, particularly in marginalised urban settings.

## Supporting information

S1 Table Description of participants’ health seeking drivers, pathways and outcomes. (DOCX)

S1 Text Interview guide for narrative interviews with people living with Non communicable diseases. (DOCX)

S2 Text A guide describing the application of COREQ guidelines within this study. (DOCX)

S1 Checklist An Inclusivity questionnaire detailing ethical approvals, community inclusion and informed consent. (DOCX)

## Notes

Drug peddlers in Sierra Leone are informal healthcare providers known for selling medicines to clients particularly in areas healthcare services are not easily accessed like in informal settlements. Many of these providers are unlicensed.

Pan-body houses are low-cost houses built typically in informal settlements using corrugated iron sheets and sticks as wells and for roofing. These houses are used as temporary or permanent housing solutions for low-income earners. They are exposed to risks due to the poor-quality materials.
