# Supplementary material for: Intersectional experiences of non-communicable diseases and health seeking strategies in informal settlements in Freetown, Sierra Leone
Source: PLOS Glob Public Health. 2026 Jul 1;6(7):e0005263. doi: 10.1371/journal.pgph.0005263 (PMC13322540; doi:10.1371/journal.pgph.0005263)
Supplement: S1 Table — (DOCX) [file pgph.0005263.s001.docx]

**S1 Table: Health Pluralism and Syncretic Health Seeking Patterns by Women and Men living with NCD Conditions**

| **Illustration key - Colour codes and diagrams representing NCD conditions and providers** | | | |
| --- | --- | --- | --- |
| **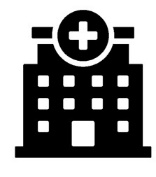**  Formal healthcare  provider (eg private & public clinics, hospitals &  pharmacies) | **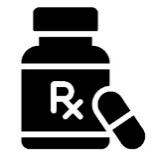** Drug peddler  (unlicensed drug seller) and authorized paramedics and  nurses providing  home-based care | **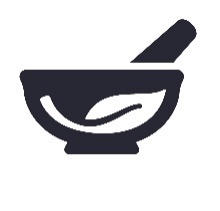**  Self-care using  traditional remedies from herb sellers | **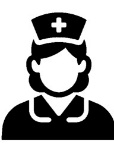**Private nurses providing care to patients at home |
| **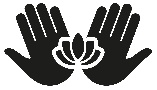** Spiritual healers providing care through spiritual   healing | **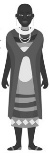**  Traditional healers / herb sellers | **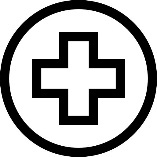**  Self-care practices using medicines from the pharmacy | 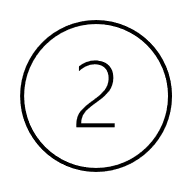**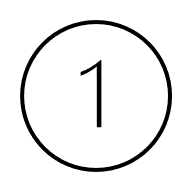**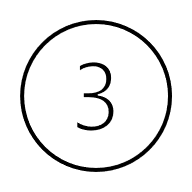  Number of interviews with participants, represent care sought  before each interview  phase |
| Non-NCD conditions | | NCD conditions | |
| **NCD type** | | | |
| Disability related to stroke | Hypertension | Diabetes | Co-morbidities of diabetes and hypertension |

| **Participant codes & pseudonyms** | **NCD type** | **Gender: Women’s Care Seeking Journeys** | **Analytical perspectives of  care seeking** |
| --- | --- | --- | --- |
| **Isata: CBY-STK-F** | **Disability related to stroke** | Isata first visited a few private clinics for numbness in her feet and severe cold severely affecting her mobility, which she attributed to stroke. She was diagnosed with and treated for malaria, typhoid and cold. Her condition did not improve following these treatments. Her family then moved her to the village to be treated by a traditional healer, and she felt a little relief without full recovery. She returned to Freetown and made further visits to private hospitals because of the cold and numbness. She was diagnosed with ulcer, which she attributed to the excessive use of painkillers in the past to self-treat pain and cold (Interview 1) Still convinced about having stroke due to persistent difficulty to walk (and observing similar symptoms from people with stroke in the community), Isata visited a government hospital and was diagnosed and treated for low blood pressure. She felt better at this point but had not recovered. She then decided to seek help from a traditional healer, but her cold got worse, further affecting her mobility due to the rubbing of herbs on her body, soaked in cold water. Observing her frailty, her church pastor with a small congregation organised a few prayer sessions at her home which made a marked improvement but still helping her to walk (Interview 2). Following these interactions, she said she was taken to a referral hospital. She said she was not formally diagnosed with stroke but was asked to participate in physiotherapy sessions. She experienced some improvement but did not last long as she dropped out of the physiotherapy due to high costs of care including transportation. She now buys medicines from a pharmacy for the self-treatment of pain and cold (Interview 3) | Sysncretic pracices were oberceverd due to the severity of illness and the lack of collaboration and referral pathways among diverse providers. Thus, limited healthcare provider collaboration can negatively impact continuum of care and patient outcomes, including slow recovery and the likelihood of catastrophic spending |
| **Illustrations of Patient/Provider Interactions** | 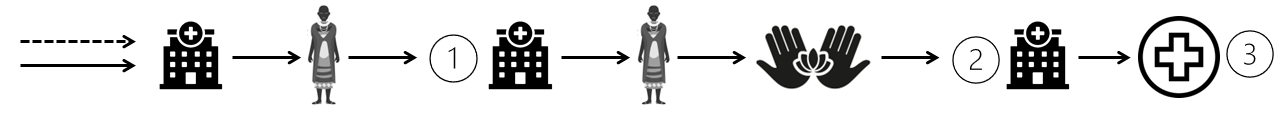 | | |

| **Participant codes & pseudonyms** | **NCD type** | **Gender: Women’s Care Seeking Journeys** | **Analytical perspectives of  care seeking** |
| --- | --- | --- | --- |
| **Hannah: DZK-STK-F** | **Disability related to stroke** | Hannah was feeling well until one night when she collapsed. She was diagnosed with stroke and treated in the hospital, but with no improved outcome (interview 1). She participated in physiotherapy sessions, which showed some improvement. However, she dropped from the sessions due to the lack of money related to direct costs of care and transportation. While at home, she buys painkillers and ointments for cold and pain from drug peddlers, with limited improvement in outcomes. Hannah therefore turned to spiritual healing sessions at an evangelical church about 2 kilometers from her hilly residence to treat stroke. She experienced some improvement but also dropped out of the spiritual healing sessions due to physical mobility and transportation challenges (Interview 2). She now stays home and seeks care from drug peddlers as the only available option to treat stroke-related pain and cold (Interview 3) | Late diagnosis of illness associated to limited prior interactions with the formal health system due to increased access barriers leads to disease progression and chronicity. Catastrophic spending also discourages care seeking, leading to uncertainty in treatment pathways and the worsening of patient outcomes. |
| **Illustrations of Patient/Provider Interactions** | 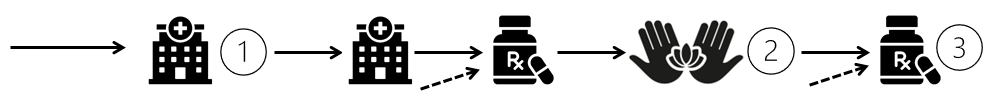 | | |

| **Participant codes & pseudonyms** | **NCD type** | **Gender: Women’s Care Seeking Journeys** | **Analytical perspectives of  care seeking** |
| --- | --- | --- | --- |
| **Yeanoh: CBY-HPT-F1** | **Hypertension** | Yeanoh had been sick with multiple episodes of ill health. Her usual complaints were severe headaches, blurry vision, and fatigue, but she did not go to the hospital. When she got worse and taken to the hospital, she was diagnosed with malaria and typhoid. She was treated to a government hospital for further care but refused to go for fear of being diagnosed or admitted for a severe illness. Instead, she was treated by a nurse at home for elevated blood pressure. She is also self-treated with ginger and honey mixture from herb sellers. Flowing its use, she asked a nurse to check her blood pressure which she said was good (Interview 1) She also checked her pressure with drug peddler who had a blood pressure machine for a small fee. She then continued to self-treat with ginger and honey mixture for high blood pressure (Interview 2) Due to the lack of money to seek hospital care, Yeanoh applied self-care using old prescription cards to buy hypertensive medicines from the pharmacy and using ginger and honey mixture to manage her high blood pressure. While the outcome was good, she was advised by her nurse to use the sparingly to prevent low blood pressure (Interview 3) | Treatment avoidance due to fear of fear of managing longterm or chronic illness, or negative prior experience with the formal health system was evident, leading to the choice of ignorance about health status. Treatment by familiar providers (e.g. nurses at home and drug peddlers) was also a strategic choice as this was thought to bring comfort through provider availability to provide prompt care and health |
| **Illustrations of Patient/Provider Interactions** | 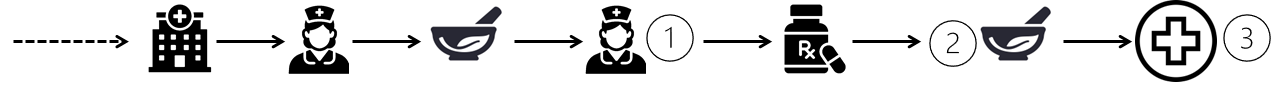 | | |

| **Participant codes & pseudonyms** | **NCD type** | **Gender: Women’s Care Seeking Journeys** | **Analytical perspectives of  care seeking** |
| --- | --- | --- | --- |
| **Margaret: CBY-HPT-F2** | **Hypertension** | Margaret has collapsed twice in the last three years. Her ailment is related to frequent heartbeats, fatigue and headaches. During the first episode, she committed to prayers and the use of medicines from drug peddlers for her recovery. Following this, her friend recommended honey and garlic to speed up her recovery. She, however, transitioned to honey and lime as the use of garlic caused discomfort, including chest burns. In her latter episode of collapse, she was taken to the hospital where she was diagnosed and treated for hypertension. However, she was unable to make the routine follow-up visits to the hospital due to the lack of money. Instead, she buys hypertensive medicines from the pharmacy to treat herself (Interview 1). Margaret continued to buy hypertensive medicines at a pharmacy to treat herself, skipping follow-up visits to the hospital due to the lack of money (Interview 2). She now uses honey and lime with hot water to treat hypertension. Outcome from this was reported to be good as she continued taking the remedy (Interview 3) | Interactions with formal healthcare systems are influenced by chronicity, knowledge about therapy management through self-care and the involvement of social networks in recommending treatments they may have tried themselves. Understandings about disease symptoms were vital in shaping oscillation among providers |
| **Illustrations of Patient/Provider Interactions** | 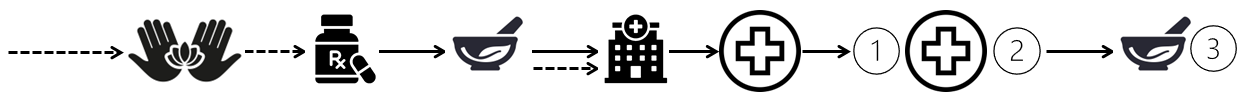 | | |

| **Participant codes & pseudonyms** | **NCD type** | **Gender: Women’s Care Seeking Journeys** | **Analytical perspectives of  care seeking** |
| --- | --- | --- | --- |
| **Mariama: MYB-HPT-F** | **Hypertension** | Mariama was first hospitalised over a decade ago following pregnancy related complication. She experiences numbness in her arms and limbs, together with pain in her neck. She became ill several times and was diagnosed during hospitalisation. She bought medicines for hypertensive (and non-hypertensive) conditions from the pharmacy to self-treat (Interview 1) She fell ill due to an intestinal condition, constraining defecation and causing a bloated abdomen. She was treated by a traditional healer for the condition which, she attributed to a witch rope placed on her waistline by a witch. The treatment involved chewing herbs and ‘‘smoking of the body’. It also involved boiling herbs and placing them in front of her, with her head covered under a blanket to sweat out the sickness. Following the intensive treatment, Mariama purchased medicines from the pharmacy to deal with the minor pain that had remained in her stomach after the witch rope was removed. She was also treated with drips by a nurse at home to rehydrate her due to excessive sweating from the smoking ritual. The nurse also treated for hypertension (Interview 2) Mariama now feels relatively better, so she buys painkillers from the pharmacy for self-care related to pain believed to be related to the witch rope removed from her stomach (Interview 3) | Health beliefs regardng disease sysmptoms and perceptions of providers were critical in influencing how people select providers and navigate them. This was evident in the way providers were selected for specific NCD and non NCD conditions |
| **Illustrations of Patient/Provider Interactions** | 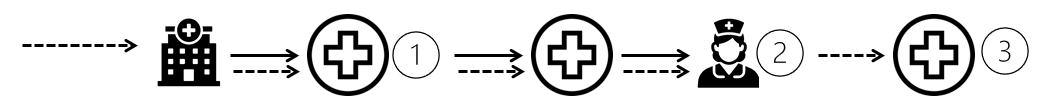 | | |

| **Participant codes & pseudonyms** | **NCD type** | **Gender: Women’s Care Seeking Journeys** | **Analytical perspectives of  care seeking** |
| --- | --- | --- | --- |
| **Salmata: DZK-DBT-F** | **Diabetes** | Salmata had been sick for over a decade with uncertainties of proper diagnosis. She was then diagnosed at a private hospital and was advised to make follow-up monthly visits. However, when she does not have money, she uses old prescription cards to buy her diabetic medications from a local pharmacy to treat herself. Her daily routine is taking one diabetes tablet (metformin) a day if her sugar level is low, and two when it’s high, although she is worried that the medication makes her body pale and emaciated, resulting from excessive weigh loss. For this reason, her sister-in-law (who is diabetic too) has convinced her about taking bitter roots (Muru and Gbangba) to reduce her blood sugar. She was convinced about the herb as her sister-in-law who uses it remains rounded despite living with diabetes for over five years (Interview 1) Salmata was admitted at the hospital after she fell ill with malaria and diabetic related symptoms (Interview 2). Not having money to go to the hospital, Salmata buys diabetic medicines from old prescriptions. She then uses newly acquired blood sugar machine to test her sugar level at home. She was concerned about excessive weight loss from the use of diabetes medications. She decided to branch out to bitter roots, following which she checks her pressure using the sugar machine at home with the help of her daughter who is a nurse (Interview 3) | Financial barriers, side effects and unsatisfactory treatment increase uncertainties about recovery. Even when formal diagnosis is acquired, self treatment becomes internalised as critical part of therapy managemnt as this involves close social networks whose experiences as patients and providers aid the process of internalising treatment options including self care. |
| **Illustrations of Patient/Provider Interactions** | 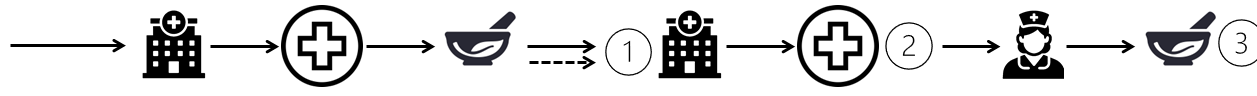 | | |

| **Participant codes & pseudonyms** | **NCD type** | **Gender: Women’s Care Seeking Journeys** | **Analytical perspectives of  care seeking** |
| --- | --- | --- | --- |
| **Mbalu: CBY-DBT-F** | **Co-morbidities of diabetes and hypertension** | Mbalu was sick with body pain, irregular heartbeats, frequent urination, and severe headaches. However, it took some time to secure a clear diagnosis. She fainted one evening while in bed and was taken to the hospital and was diagnosed with diabetes and hypertension. She takes prescribed medicines from the doctor twice daily following routine checkups. However, she finds it extremely difficult to meet her monthly follow up schedules due to lack of money (Interview 1) She missed her last schedule due to lack of money. She is treated by a nurse at home for malaria and followed by self-treatment with hypertensive medicines from the pharmacy. She occasionally uses herbs to reduce her blood sugar and blood pressure (Interview 2) She buys medicines from the pharmacy using old prescription cards for self-treatment of hypertension and diabetes (Interview 3) | Despite the repeated occurrences of ill health, securing a proper diagnosis is often a long journey. The journey through formal care seeking therefore begins only at the latter stages of ill health. For example, even after diagnosis, self-care remains a critical part of the treatment journey, due to entrenched barriers to the formal care system. |
| **Illustrations of Patient/Provider Interactions** | 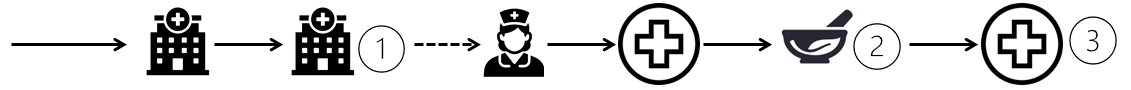 | | |

| **Participant codes & pseudonyms** | **NCD type** | **Gender: Women’s Care Seeking Journeys** | **Analytical perspectives of  care seeking** |
| --- | --- | --- | --- |
| **Nancy: DZK-HPT-F** | **Co-morbidities of hypertension and diabetes** | Nancy had been sick for many years. She had prolonged periods of stress about childlessness due to familial and social pressures. She was homeless and lonely following the death of her husband and the lack of support from family. When she got sick and taken to the hospital, it was abdominal pains related to fibroid which led to surgery. It was during this period that she was diagnosed with hypertension and comorbid diabetes. She buys hypertensive medicines from the pharmacy to treat herself. She also uses honey, garlic and lime mixture to reduce her blood pressure (Interview 1). She continues to buy blood pressure medicines from the pharmacy using old prescription cards. She had not taken the medicines lately as she had no money to replenish the medicines from the pharmacy (Interview 2). She bought some hypertensive medicines from the pharmacy using old prescription card. She also bought some medicines from a drug peddler for cold and pain (Interview 3) | The intersections of personal challenges, and limited support systems make diagnosis and treatment care seeking a temporary journey, prioritising the everyday challenges such as housing, and food. As such, healthcare only becomes a priority when it becomes a crisis. |
| **Illustrations of Patient/Provider Interactions** | 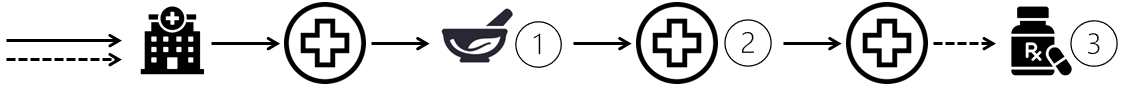 | | |

| **Participant codes & pseudonyms** | **NCD type** | **Gender: Women’s Care Seeking Journeys** | **Analytical perspectives of  care seeking** |
| --- | --- | --- | --- |
| **Sarah: MYB-DBT-F** | **Co-morbidities of diabetes and hypertension** | Sarah has faced multiple episodes of ill health over a decade causing her to collapse. Her first episode of collapse was treated by a traditional healer with a satisfactory outcome. She was diagnosed with diabetes at a private hospital in Freetown after her children brought her to Freetown due to lack of medical care in her home district in the provinces. She receives hypertensive and diabetic medicines from the pharmacy to treat herself. She also uses bitter roots to reduce high blood sugar (Interview 1) Nancy visited a private doctor who checked her blood sugar and blood pressure, and prescribed medicines for both conditions. She however continues the use of moringa, and mango leaves to reduce high blood pressure (Interview 2) She purchases diabetic and hypertensive medicines from the pharmacy using old prescription card. She also takes some herbs including boiled mango, guava and moringa leaves for both hypertension and diabetes. She takes traditional medicine in the place of ‘‘English medicine’’ in the morning when she doesn’t have food to eat, saying that English medicine requires eating first, unlike traditional medicine (Interview 3) | In a system where healthcare barriers are huge, the design of healthe seeking pathways to meet specific healthcare needs is strategic. This signals that patients are grounded in their care seeking journeys which requires that the health system must be adaptive and responsive to understand how patients must benefit from the plurality of healthcare delivery. |
| **Illustrations of Patient/Provider Interactions** | 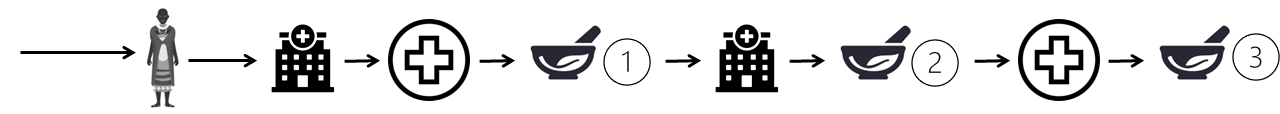 | | |

| **Participant codes & pseudonyms** | **NCD type** | **Men’s Care Seeking journeys** | **Analytical perspectives of  care seeking** |
| --- | --- | --- | --- |
| **Thaimu: DZK-STK-M2** | **Disability related to stroke** | Thaimu had been sick for many years, but it took much longer to be diagnosed. He suffers from eye impairments, severe pain in his waist, back and limbs affecting his movement. He fainted and was admitted to the hospital. He was confirmed to have had stroke and was placed on physiotherapy sessions. He was advised to make follow-up visits but occasionally makes it to the hospital due to financial limitations. He uses shea butter from herb sellers to rub on his foot and arms for pain. He also buys medicines from drug peddlers to treat the pain affecting his movement (Interview 1) He got sick again making it harder to walk and was asked to do an x-ray examination to allow further treatment. Following this treatment, he got slightly better. Thaimu buys painkillers from the pharmacy as he was advised not to buy medicines from drug peddlers during his last visit to the hospital (Interview 2) He now buys medicines solely from the pharmacy following the advice he received from the doctor (Interview 3) | The temporality of care seeking is evident here, based on financial limitations, health beliefs and proximity with alternative care systems. Care seeking is informed by the need to deal with and to seek advice (e.g. from doctors during episodic visits to the hospital) which becomes vital to individualised therapy management including the use of medications from pharmacy. |
| **Illustrations of Patient/Provider Interactions** | 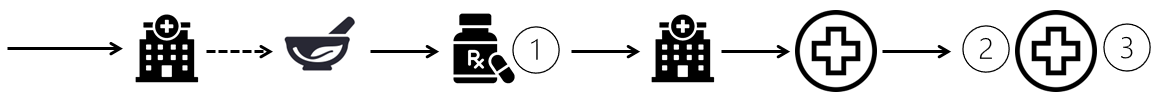 | | |

| **Participant codes & pseudonyms** | **NCD type** | **Men’s Care Seeking journeys** | **Analytical perspectives of  care seeking** |
| --- | --- | --- | --- |
| **Yamba: MYB-STK-M** | **Disability related to stroke** | Yamba has had health problems since he was young. He had been sick with severe headaches, which affected his career aspirations, as this prevented him from completing school. When his condition got worse, he was admitted to the hospital. He was confirmed to have stroke and treated for the condition. He uses painkillers and ointments (hot rubs) from drug peddlers to treat the headaches and body pain he suffers. He also uses honey to treat his frequent heart palpitations (Interview 1) He was admitted to a private hospital for stroke related conditions, including numbness in his right arms and feet. He was treated and provided with medications (Interview 2) He buys painkillers from drug peddlers to treat pain. He interacts with drug peddlers because their drugs are easily available and cheaper. He uses also honey to help manage his frequent heart palpitations (Interview 3) | Yamba’s story reflects the impact of chronic diseases that can have long term impacts on social mobility and the ability to respond to recurrent health crises. Adequate response to protracted illness was often not linear, due to financial barriers which meant that patients adapt to diverse and alternative means of accessing care including traditional remedies and medications from drug peddlers |
| **Illustrations of Patient/Provider Interactions** | 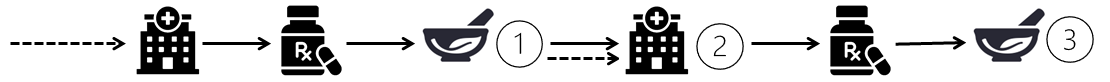 | | |
|  |  | | |

| **Participant codes & pseudonyms** | **NCD type** | **Men’s Care Seeking journeys** | **Analytical perspectives of  care seeking** |
| --- | --- | --- | --- |
| **Sulaiman: MYB-HPT-M** | **Hypertension** | Sulaiman experienced a lengthy period of ill health, without proper diagnosis and care. His usual complaint was fatigue, cold and cramps in his legs. He sought care from traditional healers for a long time, believing he was bewitched, which generally had unsatisfactory outcomes. He also bought painkillers from drug peddlers which provided temporary relief. He was later diagnosed with hypertension following previous unsatisfying diagnosis at various hospitals, some of which only treated him for severe cold (Interview 1) Sulaiman visited the hospital to check his blood pressure and to seek advice. He administered medicines from the hospital following his routine checkup (Interview 2) He made other follow up visits to the hospital, and his blood pressure was reported to be good (Interview 3) | Healthcare experiences including unsatisfactory diagnosis and treatment can challenge previously held beliefs about providers. Sulaiman’s negative experience with traditional healers and drug peddlers brings a radical shift in his belief to the formal care system. Despite his initial uncertaintty about diagnosis and high costs, he decided to stick to the formal care system due to his perceived cumulative and long-term health benefits |
| **Illustrations of Patient/Provider Interactions** | 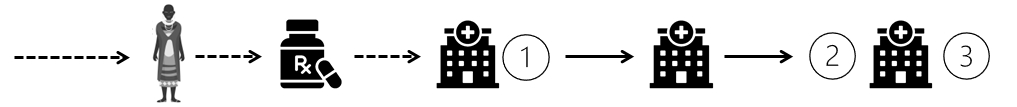 | | |

| **Participant codes & pseudonyms** | **NCD type** | **Men’s Care Seeking journeys** | **Analytical perspectives of  care seeking** |
| --- | --- | --- | --- |
| **Edward: CBY-STK-M** | **Disability related to stroke (with comorbid hypertension)** | Edwad collapsed and was admitted at the hospital. He was treated and discharged, after he felt better. Following this he was given follow-up treatment by a nurse at home. Despite his partial recovery, he could not walk by himself. He buys painkillers from a drug peddler for the pain in his limbs (Interview 1) He combines treatment from a nurse and painkillers from drug peddlers which has brough a slight relief as he can walk a bit now though slowly. The injections from the nurse have made him urinate freely, which was difficult in the last one month (Interview 2) He continues to buy medicines from a certain drug peddler because of his perceived good knowledge in treatment and counselling. He also trusts the nurse because of the health advice she provides and his continued availability for advice (Interview 3) | Edwards’ story reflects agency and the internalisation of aternative health seeking through the enactment of improved patient/provider relationship built on trust. He leverages on this relationship to improve his health awareness to help him respond to different episodes of crises, which has limited his interaction with the formal healthcare delivery system |
| **Illustrations of Patient/Provider Interactions** | 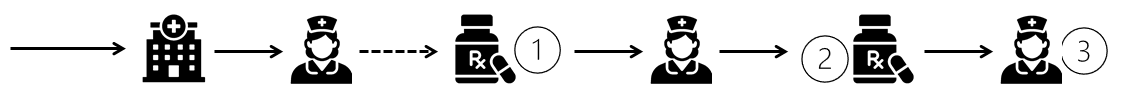 | | |

| **Participant codes & pseudonyms** | **NCD type** | **Men’s Care Seeking journeys** | **Analytical perspectives of  care seeking** |
| --- | --- | --- | --- |
| **Abdulai: DZK-STK-M1** | **Disability related to stroke (with comorbid hypertension** | Abdulai has been getting sick but preferred not to go to the hospital. He believed he could be adequately treated by a nurse at home without necessarily going to the hospital. He had severe pains in his limbs and his back, limiting his movement. He was told he had hypertension by a nurse at home, who treated him for the condition. He also buys painkillers and menthol-based gels (hot rubs) for pain (Interview 1) Abdulai was treated by his nurse at home who regularly checks his pressure and treats him for hypertension (Interview 2) He receives routine visits from his nurse to check his pressure and advise him on how to improve his blood pressure. He is treated for pain by drug peddlers for pain. His preference for drug peddlers is that they provide proper instructions to him, and their drugs are cheaper (Interview 3) | Treatment avoidance from the formal health system is also observed with Abdulai which is informed by trust in a provate provider who is more familiar with his situation and likely to bring comfort and assurance. |
| **Illustrations of Patient/Provider Interactions** | 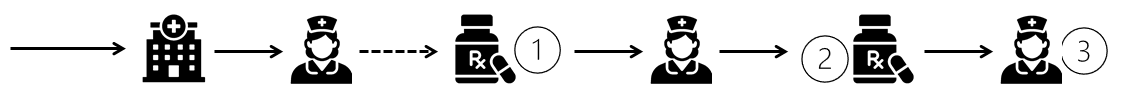 | | |

| **Participant codes & pseudonyms** | **NCD type** | **Men’s Care Seeking journeys** | **Analytical perspectives of  care seeking** |
| --- | --- | --- | --- |
| **Ishmail: MYB-DBT-M** | **Diabetes (with comorbid hypertension)** | Ishmail had been sick for several years, facing frequent headaches, fatigue, neck and back pains, and blurry vision. He was admitted to the hospital after he passed out. He was confirmed to have had comorbid diabetes and hypertension. He was eventually treated and discharged (Interview 1) Ismails was again taken to the hospital after he collapsed. He reported pains in his neck, veins and muscles, together with headaches. He was treated with hypertensive medications. To feel ‘normal’, Ishmail boils herbs, including mango, guava and moringa leaves to treat diabetes and hypertension. His decision to use herbs is based on affordability and efficacy (Interview 2) Ishmail still boils herbs (mango, guava and moringa leaves) to treat his comorbid diabetes and hypertension. He also dries some herbs into powdered forms to drink them as herbal tea (Interview 3) | Knowledge about therapy management (e,g.) using traditional remedies and the understanding of disease symptoms triggers a sensse of mastery of treatement and response to critical red flags whenever they occur. |
| **Illustrations of Patient/Provider Interactions** | 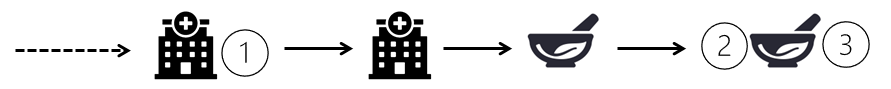 | | |

**Description of table:** The table describes health seeking in informal settlements. It reflects the intersectional nature of this study which explored the complex and overlapping drivers of health seeking in marginalised urban settlements. To enhance more clarity on the interpretation of the table and the core ideas contained within, colour codes were used to describe the types of NCD conditions experienced by women and men (See table below). The colours on the bottom left represent the NCD conditions that this study focused on, which include disability related to stroke, hypertension, diabetes and comorbidities related to the three conditions mentioned above. As part of efforts to show the intersectional drivers of health seeking, participants were further divided into gendered categories to understand the nuanced health seeking journeys of women and men. This aligns with the inter and intra-categorical approaches applied within this study which explores how social disadvantages (e.g. differences within and across gender groups) shape health outcomes, which tend to be overlooked within health systems and care delivery.

To further illustrate how patients oscillate between diverse providers, dotted arrows were used to show care seeking for non-NCD conditions such as malaria, intestinal and other febrile related conditions, while the solid arrows represent NCD conditions. These patterns indicate the temporality of care seeking informed by disease severity, recurring symptoms and financial barriers. The patterns also reveal patients’ unending anticipation for improved access to care and improved outcomes. The observations about care seeking were made over a period of twelve weeks with three interviews with each of the participants over this period (See numbers in the diagrams showing timescales for interviews).

**Description of providers:** Animated pictures were used in the treatment diaries to describe the different providers offering care to people living with NCDs (See diagrams in table). To further illustrate the complexity in seeking care within a pluralistic care system, the diagrams have been applied analytically to demonstrate the temporality of care seeking, and the constant navigation of the arbitrary care continuum. The providers in the table include formal providers (e.g. private and public health centres, hospitals and clinics), who function within recognised clinical settings. Drug peddlers included unlicensed drug sellers selling medicines and providing treatment for patients living with NCDs and other kinds of ailments. Traditional healers and herb sellers included providers offering treatment and selling various kinds of herbs, including bitter roots such as ‘‘Gbangba’’, Muru and guava leaves which are believed to be effective for the treatment of hypertension and diabetes. Spiritual healers (mostly from evangelical churches) also provide healing using divine or spiritual healing powers for the treatment of conditions such as stroke. Pharmacies selling medicines and nurses providing care within private homes are also described within the table. While nurses were often described by participants as qualified and experienced, the provision of care outside authorised clinical settings may not sit adequately within the description of formal care. This clarification is important within the context of the Sierra Leonean Health System (and other pluralistic health systems in LMICs) which frown at providers offering care at home due to quality-of-care concerns. Thus, in the context of private nurses providing treatment at home, it may not be strictly fall within the remit of formal healthcare in Sierra Leone, as formal care is prescribed more in the context of healthcare in a clinical setting. This description requires further scrutiny in the LMIC settings to provide more clarity on the role of clinicians in community settings particularly in the context of constrained chronic care management. Pharmacies are also described in the table who provide medicines over the counter to patients. While pharmacies and drug stores are authorised within the formal healthcare system, the kinds of care described by patients may not entirely fall within the remit of formal care as it often involved self-care by patients who either bought or administered care without prescription or clinical supervision.

**Description of care seeking patterns**

Syncretic seeking practices were observed among women due to severity of illness, financial barriers, and uncertainties about diagnosis leading to unsatisfactory treatment outcomes. Limited collaboration among formal and informal providers, evident by the absence of proper referral pathways further complicated health-seeking journeys. Women living with disability from stroke, interacted with physiotherapy services to enhance improved mobility, following shocks such as collapse, while drug peddlers treated pain and cold, using painkillers and hot rubs. These remedies were sometimes combined with prayers and healing sessions, particularly when recovery from traditional and formal healthcare providers was observed to be slow. The lack of proper referral system often disrupted the continuum of care, increasing the likelihood of catastrophic spending, slow recovery and overall negative patient outcomes. These trends caused desperation, and hopelessness, particularly for women and men living with disabilities. Moreover, limited access to formal healthcare delivery, influenced by perceptions of high costs of care and service inefficiency contributed to late diagnosis, leading to disease intensification and chronicity.

For some women with hypertension, syncretic seeking from formal and informal providers such as ‘‘drug peddlers’’ was observed in response to specific symptoms such as headaches and body pain. While care seeking formal providers was recognised as important in ensuring overall health benefits, treatment avoidance was observed by some to enact ignorance about their illness status, to avoid the consequences of high financial costs associated with long-term illness, and negative prior experience with the formal healthcare delivery. Instead, the choice of treatment by a familiar provider such as private nurses at home or drug peddlers was intuitive as this was believed to bring comfort, and health advice. Furthermore, health beliefs regarding disease symptoms and perceptions of providers were critical in how healthcare decisions were made. This knowledge was vital to whether patients selected providers to deal with specific conditions such as drug peddlers for the treatment of pain and headaches; pharmacies for self-treatment of hypotensive conditions or herbal remedies to manage high blood pressure; and hospitals to manage disease intensification. For women with diabetes, care seeking from formal providers was somewhat consistent due to the need to constantly abreast themselves with their health status. Care seeking from formal providers by some women was also informed by relatively satisfactory outcomes, despite the side effects such as excessive weight loss. Self-care practices using medicines from the pharmacy were also identified as critical part of therapy management, due to financial barriers, unsatisfactory outcomes and the involvement of close social networks in therapy management.

For women with comorbidities of diabetes and hypertension, care seeking journeys were not always linear. These journeys were informed by the intersections of personal challenges, and limited support systems making diagnosis and treatment a temporary journey, prioritising the everyday challenges such as housing, and food. Even after diagnosis, self-care remained a critical part of the treatment journey, making the prioritisation of formal care seeking more towards crisis management.

Similar patterns of health seeking were observed among men. For men living with disability related to stroke, the temporality of interactions with formal healthcare system was evident, based on financial limitations, health beliefs and proximity with alternative care systems. More care seeking was from drug peddlers, and treatment from private nurses at home. treatment seeking from formal healthcare providers was therefore informed by crisis and the obtainment of critical health advice which aided the process of self-therapy management. Desperation and uncertainties about recovery by men living with disability from stroke reflected the framing of the human model of disability which speaks to the long-term impact of chronic diseases on social mobility and the ability to respond to recurrent health crises. As such adequate response to protracted illness journeys in the treatment of disability related to stroke was often not linear.

While men with diabetes, disability related to stroke (and comorbid hypertension) interacted with formal providers in response to different episodes of disease intensity, they showed strong agency towards improving their interactions with alternative providers (e.g. drug peddlers, herb sellers and private nurses to improve their response to their daily health problems. For Ishmail who lives with diabetes (and comorbid hypertension), knowledge of therapy management using traditional remedies (e.g. guava, moringa and mango leaves) was vital in enhancing his illness and treatment journeys. The same applies to Edward and Abdulai who have created beneficial relationships with nurses and drug peddlers to enhance timely access to care, and critical health advice (See table above).

However, one participant who identified with hypertension (Sulaiman) provides an interesting illustration of shifting health beliefs. He lack of trust in traditional healers and drug peddlers was due to repeated treatment failures. The shifting of trust towards the formal care system, was due to the cumulative and long-term health benefits (despite the initial uncertainties about diagnosis and high costs).
