# Supplementary material for: Intersectional experiences of non-communicable diseases and health seeking strategies in informal settlements in Freetown, Sierra Leone
Source: PLOS Glob Public Health. 2026 Jul 1;6(7):e0005263. doi: 10.1371/journal.pgph.0005263 (PMC13322540; doi:10.1371/journal.pgph.0005263)
Supplement: S1 Text — (DOCX) [file pgph.0005263.s002.docx]

**S1 Text: Narrative Interview Guide**

**Participant Characteristics**

Interviewee ID………………………………………………………………………

Date of Interview: ……………………………………………………………………

Gender: ………………………………………………………………………………

Age: …………………………………………………………………………………

Type of NCD: ………………………………………………………………………

**Introduction**

Narrative interviews will be done to understand the care seeking strategies of people living with chronic health conditions (diabetes, hypertension and stroke) in informal settlements in Freetown, Sierra Leone. We are interested and open to them telling us about anything they think is important about health over the course of their life. You should therefore explain to the interviewee that in this interview we want to hear them talk about their life, and the health events that have shaped their lives. This involves their health seeking preferences and how they are influenced by personal and social circumstances. A selected cohort of participants with chronic health conditions will be interviewed three times with a six-week break between each interview. The interviews will be divided into two parts as described below:

**Phase one**

**First visit**

- **Life histories:** The first meeting will explore patients' life histories regarding family history, health and social life and how these have shaped their current life. It will also explore everyday challenges such as the environmental problems, age and gender and how these link to health problems and seeking strategies. The life histories will also find from patients about their hopes for the future.

**Phase two**

**Second and third visits**

- **Health seeking beliefs and strategies:** The second and third visits will focus on health seeking strategies generally. This will be explored within the health belief model which will find out why explore motivations for health seeking decisions. Seeking choices will be explored to understand how health beliefs, quality of care, and intersecting social vulnerabilities influence health seeking. Using visual diaries, participants will be asked prior to the interviews to indicate where they have sought care and for what reason. The pictures will aid them to identify the different care providers (eg. Traditional healers, allopathic providers etc.) they visited, and will provide the basis for the discussions that follow.

**Participant Life History Sessions**

**First visit**

*During this visit, try to establish rapport with the interviewee. This is a critical stage of the interview phase as it intends to build trust for follow-up meetings. Ensure that the participant is made comfortable and fully aware of the purpose of the interview and how many times they will be contacted. Once confidence is built, it is hoped that interviewees will be comfortable to articulate their personal life trajectories and their experiences with health and health seeking.*

***Opening question:*** Thank you for agreeing to take part in this interview. Can you please describe to me your life events? I would like you to draw me a line, starting with birth unto the current stage. On the line, I would like you to mark off key life events which I will then ask you to describe to me”. These may include things such as; family life, education, illness or death within the family, marriages, births, etc.

Make the necessary probes regarding the different key events as the participant begins to explain

**Probes**

- And then what happened?
- Who did that? Who was responsible for this?
- Did you go anywhere or ask anyone for help? Who? Where?
- How did you and/or your family manage during this time?
- What was happening in your life before this?
- What impact did this have on your life? What did it do to your life?
- How has your life been since that that time/this event?
- How do you protect yourself and your family from illness/health problems?

**Final related questions**

- What is the general state of health service provision and access in this community?
- What are the factors that impact your health and access to health (probe…..are these related to environmental, social and political factors?
- How does seasonality (rainy season/dry season) affect your health and influence your health seeking decisions?
- What do you think must change to improve your access to healthcare?

**Second visit**

*This session draws from the last contact by finding out from interviewees their current state of health and whether they have sought help from any provider since that time. It also tries to establish links between beliefs and health seeking strategies. The visual diaries template will be used at the beginning of the interviews to find out from interviewees where they have sought help since the last visit and for what condition. They will fill out the appropriate boxes as necessary to understand the outcome of each visit and how that will shape their decisions about health seeking in the future.*

| ***Health provider*** | ***Symbol of health provider*** | ***Health condition for which you visited a provider*** | ***Outcome: How many visits in the last six weeks? Did you access the care you wanted? Did you get healed from this visit? Any challenges?*** | ***Overall, were you satisfied with the service you received? Why?*** |
| --- | --- | --- | --- | --- |
| Traditional healers/herb sellers | **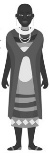** |  |  |  |
| Spiritual healer | **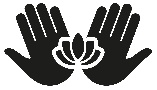** |  |  |  |
| Drug peddler/unlicensed (pepe doctor) | **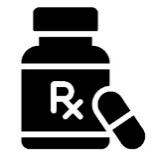** |  |  |  |
| Private nurses providing care at home | **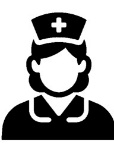** |  |  |  |
| Formal healthcare provider (public/private clinics, hospital & pharmacies) | **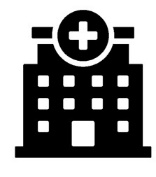** |  |  |  |
| Selfcare with medicines from pharmacy/drug store | **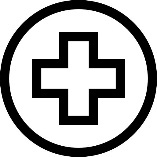** |  |  |  |
| Self-care using  traditional remedies from herb sellers | **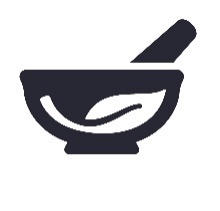** |  |  |  |

- How has your health been since the last visit?
- Have you visited any healthcare provider since that time? (make further prompts why they visited the provider they named)
- Where do you regularly seek help when you are sick?
- What are the different health providers in this community?
- Which of the health providers do you prefer and why? Is this influenced by quality or cost of care?
- What factors inform your choice of healthcare providers you seek help from? (probe…..cultural? religious? Belief?
- Are there any current personal circumstances that influence your decision about where to seek healthcare? ( probe…..eg. Age, gender, health/social status?)
- How does your family, community etc. support you to have adequate access to healthcare?

**Third visit**

*At this stage, try to synthesize key learnings from discussions in prior visits by linking the participants’ personal circumstances with their health seeking strategies. Try to find out if there is anything they would like done differently to improve their access to healthcare.*

- How has your health been since the last time we spoke?
- Have you sought help from a heath provider since our last meeting? From whom and why?
- What personal circumstances affecting your health and access care that you think must be addressed?
- What do you think about the state of healthcare provision in this community? Who do you think is contributing most to improve access to care for people with chronic health conditions
- What do you think must be done to improve access to healthcare for people with chronic health conditions in this community?
- What are your hopes for health service provision and access in this community?
- Is there anything else you what to add to all what you have said? Is there any question you may want to ask?
