# Supplementary material for: Intersectional experiences of non-communicable diseases and health seeking strategies in informal settlements in Freetown, Sierra Leone
Source: PLOS Glob Public Health. 2026 Jul 1;6(7):e0005263. doi: 10.1371/journal.pgph.0005263 (PMC13322540; doi:10.1371/journal.pgph.0005263)
Supplement: S2 Text — (DOCX) [file pgph.0005263.s003.docx]

**S2 Text: Supporting Document for PLOS Global Public Health**

**COREQ Guidelines**

As requested, we have ensured that the manuscript aligns with the COREQ guidelines to enhance transparency and rigour. We have maintained a high level of accuracy and transparency by addressing the checklist’s three primary domains: 1). Research Team and Reflexivity 2). Study Design, and 3). Analysis and Findings

| Research Domains | Sub domains | Description of activities |
| --- | --- | --- |
| 1) Research team and reflexivity | Personal characteristics | The interviews for this study were conducted by the first author (AC, a Sierra Leonean national) who brought in extensive expertise in gender, health and intersectionality, coupled with embeddedness in the Sierra Leonean Health system and in informal settlements. All authors in this study are highly experienced in health systems research, particularly in fragile and conflict affected settings |
|  | Relationship with participants | While the lead researchers had no prior relationships with the participants, community-based co-researchers utilised their existing rapport and previous interactions within the community to facilitate engagement. To ensure informed and voluntary participation, all participants were provided with detailed study information. This process fostered trust between the interviewer and participants and ensured good rapport during the interviews |
| 2). Study design | Theoretical framework | This study was guided by an adaption of the Intersectional Gender Analysis Framework for Infectious Diseases of Poverty Research (WHO, 2020), which itself drew from the Intersectionality Wheel (Simpson, 2009). The framework was specifically applied to an informal settlement context to explore how gender identities interact with poverty to shape vulnerability to Non communicable diseases (NCDs) and the capacity of residents to seek early and appropriate care. |
|  | Participant selection | The study used purposive sampling to recruit 15 participants for narrative interviews, based on criteria, including type of NCD, age, gender, and place of living within the settlements. This approach was designed to achieve a diversity of participants’ characteristics and perspectives. Participants were approached by co-researchers as part of multidisciplinary research – [Accountability, Responsiveness and Equity Hub (ARISE)](https://ariseconsortium.org/learn-more-archive/arise-learning-report-4/) to strengthen equitable partnerships with marginalised urban residents. |
|  | Study setting | This study was conducted across three urban informal settlements in Freetown, characterised by spatial informality and lack of urban planning. These sites possess distinct physical features such as proximity to the seafront or steep terrain that are often overlooked regarding how they shape residents' health vulnerabilities and access to care. Specifically, the study included Cockle Bay, a seaside settlement in western Freetown, alongside Dwarzark and Moyiba, which are hillside settlements located in the central and far-eastern districts, respectively. |
|  | Data collection | Narrative interviews were conducted with 15 participants over a twelve-week period. The data collection was structured into three household visits across two distinct phases. Phase one explored participants’ narratives regarding ill-health, livelihoods, educational attainment, and household gender relations. Phase two focused on health-seeking decisions, including health beliefs, costs, and quality of care. To facilitate recall and dialogue, participants utilised visual treatment diaries to document their health-seeking journeys, preferred providers, presenting conditions, and treatment outcomes. All interviews were audio-recorded following established informed consent procedures. |
| 3). Analysis and findings | Data coding | A framework approach was inductively and deductively employed to systematically organise the data and identify emerging patterns. The analysis was guided by an initial coding framework derived from the study objectives and emerging insights from the data, which provided a structure that allowed the emergence of new themes. Primary coding was conducted by the first author (AC), with the broader research team contributing to the design of the framework and the collaborative interpretation of the data to ensure analytical rigour. |
|  | Derivation of themes | The systematic application of the coding framework facilitated the identification and refinement of core themes. These themes underwent rigorous interpretation to ensure they were seamlessly integrated into the findings, providing a cohesive narrative that directly addressed the study’s research objectives. |
|  | Software used | NVivo 14 software was utilised to organise and code the data using a case-based approach tailored for intersectional analysis. Each participant was assigned specific case attributes, including community, age, gender, and disease typology, which served as stratifiers for comparison of lived experiences across identities of people living with NCD conditions. These attributes were integrated into a framework matrix, enabling the systematic cross-tabulation of intersecting identities within rows against emerging thematic codes within columns. |
|  | Quotations presented | Verbatim participant quotations were systematically integrated across all themes and sub-themes to illustrate and substantiate findings. Each quotation was selected purposefully to preserve the specific contextual interpretations and social settings within which the codes were analysed, ensuring a transparent link between the raw data and the final analytical conclusions. |
|  | Clarity of major themes | The major themes developed from the findings were iteratively reviewed to ensure conceptual consistency with findings. This critical verification process guaranteed that the emerging themes remained robustly integrated with the intersectional framework adapted for this study. |
