# Supplementary material for: Intersectional experiences of non-communicable diseases and health seeking strategies in informal settlements in Freetown, Sierra Leone
Source: PLOS Glob Public Health. 2026 Jul 1;6(7):e0005263. doi: 10.1371/journal.pgph.0005263 (PMC13322540; doi:10.1371/journal.pgph.0005263)
Supplement: S1 Checklist — (DOCX) [file pgph.0005263.s004.docx]

S1 Checklist: Inclusivity in global research

PLOS’ policy on inclusivity in global research aims to improve transparency in the reporting of research performed outside of researchers’ own country or community and ensures that PLOS publications reporting global research adhere to high standards for research ethics and authorship. Authors of relevant research articles may be asked to complete the questionnaire below, which outlines ethical, cultural, and scientific considerations specific to inclusivity in global research. This questionnaire may be requested when researchers have travelled to a different country to conduct research, if research uses samples collected in another country, research with Indigenous populations or their lands, or if research is on cultural artefacts. Researchers travelling to another country solely to use laboratory equipment will not normally be required to complete the questionnaire. However, the questionnaire can be requested at the journal’s discretion for any submission – if you have been requested to complete this questionnaire by the PLOS journal you submitted to, please do so.

Please complete the questionnaire below and include this as a Supporting Information file with your manuscript. Note that if your paper is accepted for publication, this checklist will be published with your article in the supporting information files. Please ensure that you reference the checklist in the main body of your manuscript. We suggest adding a subsection ‘Inclusivity in global research’ to your Methods section and adding the following sentence: “Additional information regarding the ethical, cultural, and scientific considerations specific to inclusivity in global research is included in the Supporting Information (S1 Checklist)”

The questions have been designed to be applicable to a wide range of study types, and there are subsections for both human subjects research and non-human subjects research. If any of the questions are not relevant to your research, please mark them as “N/A” as appropriate.

**Ethical considerations, permits and authorship**

*This section is applicable to all research types.*

Provide details as to who granted permission and/or consent for the study to take place in the Methods section of your manuscript. This should include the names of **all** ethics boards, governmental organizations, community leaders or other bodies that provided approval for the study. If individuals provided approval refer to these people by their role or title but do not list their name(s).

Reported on page number 222 - 225: Ethics approval for this study was sought and obtained from the following national and international ethics granting institutions: 1) Approval was granted by the Sierra Leone Ethics and Scientific Review Committee (SLESRC) on 3rd August 2021 with version number 2.0 of 28 July 2021, signed by the Chair of the Ethics Committee. Approval was also granted by the Research and Ethics Committee of the Liverpool School of Tropical Medicine, (LSTM), also signed by the Chair of the Research Ethics Committee on 5^th^ August 2021 with reference number 21-043.

If there were any deviations from the study protocol after approval was obtained, please provide details of these changes in the Methods section of your manuscript.
Did this study involve local collaborators that are residents of the country where the research was conducted, or members of the community studied? If you do not have any authors from said communities, please provide an explanation for this below.

The first author of this study, AC, together with the 4^th^ and 5^th^ authors, JM, and BM are from Sierra Leone, where the study was designed and conducted, and have published widely on informal settlement vulnerabilities. In addition, this study involved local collaborators referred to as co-researchers who reside in the three study sites in Freetown. Co-researchers are community residents who are involved in research to convey the voices of residents in the design of research to improve overall health and wellbeing outcomes in marginalised spaces. Their inclusion was part of the Community Based Participatory Research (CBPR) within the [Accountability and Responsiveness in Informal Settlements for Equity (ARISE) Consortium](https://ariseconsortium.org/). Within this study, co-researchers supported the recruitment of study participants for the three rounds of interviews and participated in data collection. However, co-researchers were not included as authors as their contributions did not reach the threshold to be listed as authors, including the drafting and review of manuscript. Moreover, ethical and logistical issues including the time required to review the manuscript and having an email or ORCID address would have constrained the timeline completion of this study. However, their contributions were adequately acknowledged in this article (See acknowledgements section) and were awarded certificates for their contribution to the ARISE project.

This study undertook no activity or work that deviates from the provisions of the study protocols granted to this study.

Everyone listed as an author should meet PLOS’ criteria for authorship and all individuals who meet these criteria should be included in the author byline, rather than the acknowledgements. For further information please see the journal’s Authorship Policy.

**Human subjects research (e.g. health research, medical research, cross-cultural psychology)**

Did you obtain written informed consent from a representative of the local community or region before the research took place? How did you establish who speaks for the community? Details of written informed consent obtained from study participants should be reported separately in the Methods section of your manuscript.

Consent with local community: As part of the community entry, we established contact with community stakeholders including the community chiefs and co-researchers. As per traditions and norms in Serra Leone, we did not sign a written consent but were allowed to conduct the research after explaining the purpose of the research and its potential benefits to stakeholders. Across the study sites, community chiefs were the main contacts for the study, through the co-researchers.

Informed consent with study participants: Participants in this study provided informed consent after they were informed about the purpose of the research and its potential contribution to the strengthening of policies and interventions to improve NCD healthcare delivery for people living in vulnerable urban settlements. Participants were informed about the importance of informed consent, including their right to participate or withdraw from study without reprisals. Participants who were recruited were asked to sign a written consent form before each of the interview sessions, after providing them with sufficient information or clarifications on the consent procedures.

How did members of the local community provide input on the aims of the research investigation, its methodology, and its anticipated outcome (s)? When engaging with the local community, how did you ensure that the informed consent documents and other materials could be understood by local stakeholders?

As part of the CBPR process described earlier, the views of community members were reflected in this study through the engagement of co-researchers in the refinement of the research tool and in data collection. Co-researchers were also involved in reflexivity sessions to reflect on the data collection procedures and to cross check whether the emerging insights reflected the daily challenges faced by people living with Non communicable diseases (NCDs). The co-researchers also supported consent procedures which were preceded by the recruitment. Prior to the recruitment and consent, co-researchers provided or read out the information to participants in the information sheet to allow them to make informed decision about their involvement in the study. Beyond the consent procedures, co-researchers served as entry point to engage with community stakeholders (e.g. chiefs) to inform them about the purpose of the research.

Will the findings of the research be made available in an understandable format to stakeholders in the community where the study was conducted (e.g. via a presentation, summary report, copies of publications, etc.)? Please provide details of how this will be achieved.

Findings from this study will be presented to community and health system stakeholders after it has been submitted for publication. This will include summarising the findings to improve discussions about the challenges of living with NCDs and what individuals, households and stakeholders can do to improve access to healthcare. Copies of the summarised findings will be provided to community stakeholders while electronic copies of the study publication will be shared with health system policy stakeholders within the ministry of health and other institutions working towards health system strengthening such as NGOs. The publications will also be made available on the website of the Sierra Leone urban research centre: [www.slurc.org](http://www.slurc.org). The website is currently being updated to improve access and utilization by diverse stakeholders involved in urban knowledge and addressing heath inequities in Sierra Leone and beyond.

**Non-human subjects research using specimens/ animals collected as part of the study, or those housed in archival collections. Examples include archaeology, paleontology, botany and zoology.**

Did the permission you obtained from a local authority to perform the study include an agreement on access to outputs and benefit sharing? This may include procedures to enable fair distribution of the benefits and resources arising from the research carried out. Please include any details of Prior Informed Consent and Benefit Sharing Agreements obtained. These may be required by field-specific regulations, for example the Convention on Biological Diversity (CBD) and the associated Nagoya Protocol.

N/A

If the material used in your study was imported, please A) provide the year it was imported and B) indicate whether permits were obtained to import/export the materials used, C) provide details of any permits obtained. If this information is not available, please indicate this.

N/A

If you used archival specimens, please state how the material used in your study was acquired by the institute it is held in and provide details of any permits obtained for the original excavations/ sample collection. If this information is not available, please indicate this.

N/A

How was the potential cultural significance of the materials collected in your study to local communities considered in your research design? Were Indigenous peoples and/or local researchers and institutions involved with archaeological excavations / collection of specimens? If so, please provide a description of their involvement.

N/A

If your manuscript includes photographs of human remains please indicate whether authors obtained permission from descendants or affiliated cultural communities to do so.

N/A
