# Supplementary material for: Intersectional experiences of non-communicable diseases and health seeking strategies in informal settlements in Freetown, Sierra Leone
Source: PLOS Glob Public Health. 2026 Jul 1;6(7):e0005263. doi: 10.1371/journal.pgph.0005263 (PMC13322540; doi:10.1371/journal.pgph.0005263)
Supplement: Attachment — Submitted filename: Review assignment for PGPH-D-25-02619.docx [file pgph.0005263.s005.docx]

This manuscript is well-written and engaging, and I would like to commend the authors for presenting such rich and dense information.

Here are my comments, which I believe will enhance the content of this manuscript.

1. Page 2, line 27 (abstract section): The term "oppression" appears quite strong, and I do not find any evidence supporting it in the results section. Could the authors clarify what they mean by "oppression"? Additionally, the sentence “as gender norms and experiences of masculinities and femininities reflected the division of roles and access to resources by men and women, which in turn shaped their ability to seek early and better healthcare interventions” seems to contradict the notion of oppression if participants are able to seek early and improved healthcare interventions.

2. Page 6, line 133: typo: a dot is in the middle of the sentence. Consider removing.

3. Page 7 on selection of study participants: How did researchers identify participants based on diabetes and hypertension criteria, given that these medical conditions require a confirmed diagnosis from health professionals? Additionally, were any participants identified using a snowball sampling technique?

4. It is mentioned that this study was conducted as part of the broad ARISE project. Can the authors clarify whether the study participants were enrolled into the main project and if so, to what extent this initial involvement of the main study had impacted the data collection?

5. An important section on data collection is missing. I would like authors to include this in order to explain what specific data were collected at each round, why was it necessary to split data collection over different timepoints, where and in which language were data collected, how long lasted interviews on average, and who collected the data, any bias that may result from the way the study participants perceived the data collection team during fieldwork?

6. The data analysis section requires further clarification. It would be beneficial if the authors could elaborate on their data coding strategy. It appears that a deductive approach was employed, yet they did not explain how the coding frame was initially developed before its application to the data, nor did they specify who conducted these procedures. Additionally, information regarding the software used to facilitate the data analysis is missing.

7. The use of relevant qualitative data reporting guideline is missing. This is important to include as it will help ensure that ensure that the methods and findings are clearly and transparently communicated in the manuscript.

8. In relation to adapting the framework for Infectious Diseases of Poverty Research, it would be beneficial if the authors could clarify the specific adaptations made to the framework and how it was applied during data analysis.

9. They mentioned that participants who faced distress during the interviews were allowed to grieve and were consoled. How many participants developed such condition during interviews at which round of data collection, and to what extent did this impact on data collected?

10. This study seeks to explore the lived experiences of individuals affected by NCDs in informal settlements. However, the findings presented in the results section, particularly the initial part titled "lived experience of NCD," fall short of adequately conveying the participants' current experiences with these health conditions. Firstly, the authors include excerpts from three informants but fail to demonstrate how these accounts are representative of all study participants. Secondly, the discussion of informants' lives prior to developing their current health conditions renders the information less pertinent to the study's objectives. It would be beneficial if the authors could focus on participants current lives to discuss the intersectionality of NCDs with social, economic, and gender factors within the participants' living environments, and how these elements contribute to shaping the management of their health conditions. This way of presenting the findings restricts our understanding, as the same observation is reiterated throughout the discussion section.

11. In the context of chronic disease, it can be assumed that participants with diabetes, hypertension, or comorbidities might encounter similar yet distinct challenges. It would be beneficial for the authors to discuss the findings across these cases, highlighting both similarities and differences, and to establish a hierarchy within the data.

12. The use of terms like "people," "women," or "men" in the results section can be misleading, as they might suggest that the findings apply to the general population. Instead, it is advisable to use terms such as "informants," "study participants," or "men" or "women in this study" to ensure that the information reported is limited to those individuals who participated in this data collection.
