# Supplementary material for: Intersectional experiences of non-communicable diseases and health seeking strategies in informal settlements in Freetown, Sierra Leone
Source: PLOS Glob Public Health. 2026 Jul 1;6(7):e0005263. doi: 10.1371/journal.pgph.0005263 (PMC13322540; doi:10.1371/journal.pgph.0005263)
Supplement: Attachment — Submitted filename: Response to Reviewers_PLOS_31_01_26.docx [file pgph.0005263.s007.docx]

PLOS Global Public Health

Response to Reviewers

| Reviewer type | Comments | Response |
| --- | --- | --- |
| Reviewer # 1: General comments | The article is relevant and timely. Please use COREQ guidelines/other community guidelines for qualitative research listed on the EQUATOR network to improve the rigour and flow of the article. Additional suggestions for your consideration are listed below. | While we tried to maintain accuracy and transparency as much as we could, we initially did not follow a step-by-step COREQ reporting format. Following your recommendation, we have now reviewed the article based on the COREQ guidelines to strengthen rigour and flow. We have strengthened the methodological approach, participant selection, data collection, description of research sites, relationship with local community structures (e.g. co-researchers) and analysis of data, considering the use of software, coding procedures, design of codes and themes, adaptation of a conceptual framework, and the writing of findings. |
| Specific comments: 1 | The rationale for the article as well as research question needs to be made more explicit. Additionally, the novelty of the article is relatively unclear. | We have reflected on your comment on the rationale of the article and its novelty. Regarding the rationale, we have provided a response in lines 19 - 28(in the abstract) which argues that the intersectional inequities shaping NCDs in marginalised urban settings remain less understood and less prioritised within policy and practice, which amplifies the burden of NCDs, including reduced capacity to seek timely and appropriate healthcare. We argue therefore that policy making processes in LMICs should look beyond the disease centric approaches to healthcare delivery and instead consider the social and structural drivers that underpin these inequities. We have provided additional information about the research gap, and hence the rationale of this study in addressing this evidence gap (See lines 55 – 65 in the introduction).  We have also more clearly articulated the novelty of this article through its methodological approach which adapts the Intersectional Gender Analysis Framework for Infectious Diseases of Poverty Research to explore the lived realities of NCDs in an informal settlement context, using a narrative inquiry approach. Please find the descriptions of this approach in lines 206 – 220 in the analysis section. |
| 2 | The use of a framework devised for infectious diseases needs to be examined- why was this framework chosen? | Thank you for this important point.  The framework for Infectious Diseases of Poverty underscores the important interactions between poverty and infectious diseases that are often endemic in resource poor settings (e.g inadequate housing, healthcare and sanitation) and are too often overlooked within health policy. We therefore adapted this framework as part of our analysis to explore the many social drivers of NCDs which overlap with infectious diseases of poverty, as a good starting point to explore NCDs in informal settlements. We also explored these interactions with respect to the intensification of NCDs in informal settlements, and how it impacts the ability of affected persons to cope with the conditions and seek appropriate and early care. You can find these justifications in lines 206 – 224 in the analysis section, and 738 – 743 in the discussion. |
| 3 | Line 52-54: It may be useful to examine the cause of death in context of age; additionally, the later use of terms like young-old-middle aged are ambiguous later in the article, please provide an operational definition. | Thank you for your interest in understanding the cause of death in the context of burden of NCDs. While the cause of mortality is an important subject, we have not explored it in great depth, as it falls outside the scope of this study. This study has focused more on the lived experiences of NCDs, including gendered inequities and their influence on coping and health seeking interventions.  Regarding the use of age categories, we have reviewed the texts in table 1 to update them by indicating the age ranges of participants to address the confusion about the earlier descriptions such as ‘young’, middle-aged’ and ‘old’ |
| 4 | Line 54-57: The reason for prioritising lived experience is unclear. The logical flow establishing the rationale for this inquiry needs strengthening. | We have provided some context about the precarities of informal settlements such as poor housing and environmental conditions, and limited access to services such as healthcare, which amplify the risks of NCDs and reduced capacity to seek early and adequate healthcare (please see lines 73 – 85). We posit that this addition fits well into the argument about lived experiences of NCDs in marginalised urban settlements and the decision to focus on exploring these realities to understand the broader and multiple dimensions of exclusions that shape NCD vulnerabilities. You can find the research gap and the justification of the study in lines 55 – 65. |
| 5 | The linkages of NCDs with urbanisation need more citation and can be explored better. The authors may consider reassessing the introduction section which seems to appear definitive without providing a research gap. | We have revised the introduction section to provide context and to strengthen the linkages between NCDs and urbanisation (lines 66 – 72), and the research gaps here (lines 55 -65). We argue that NCDs, interacting with urbanisation, have added to the existing layers of urban vulnerability, partly due to the exclusion of the urban poor in the design of urban policies, which reinforce inequities, particularly in housing, and healthcare access, and expose them further to negative health outcomes. We also highlight that understanding the interactions of rapid urbanisation, poverty and the increase of NCDs is critical. To strengthen these linkages, we have identified gaps within health systems policy and practices such as the limited understanding of NCD drivers and prioritisation within policy and practice. Addressing these gaps will help to reduce the burden of NCDs and improve access to healthcare in marginalised urban areas. |
| 6 | Why is this research needed? What makes it relevant for global health? This needs to be elucidated in the text. | This study applies an intersectional lens to understand health inequities and outcomes, adapting a framework for infectious diseases of poverty to explore the relationships between NCDs, gender inequities and poverty. In applying this approach, we have highlighted the importance of addressing health inequities by understanding how social identities interact to shape coping with NCDs and health seeking. We argue that using this approach in health systems research will help policy makers in fragile health settings to design actions that will tackle health inequity, which is relevant to the attainment of the Universal Health Coverage by 2030 (Please see lines 55 - 65) |
| 7 | The use of the word "drug peddler" should be in quotes at least at first use, to ensure that it is seen in its local context. | Thank you for your suggestion. We have inserted quotes on the word ‘‘drug peddlers’ which first appeared in table 1 (See pseudonym for participant - Yamba). However, since this appearance is in the table (and some readers sometimes only refer to tables after reading the main text), we decided to use the quote in the main text in line 553. We also provided a note in line 856 at the end of the manuscript to describe drug peddlers. |
| 8 | The narrative findings are rich and well presented- the global health impact of these findings/future paths based on these findings need to be made explicit. | Thank you very much. We have provided a response with regard the global relevance of this research in comment number 6 above. |
| Reviewer # 2: General comment | This manuscript is well-written and engaging, and I would like to commend the authors for presenting such rich and dense information. | Thank you very much. We are hopeful that the manuscript will contribute to global knowledge on NCD outcomes and improving healthcare access for urban marginalised groups |
| Specific comment s:1 | Page 2, line 27 (abstract section): The term "oppression" appears quite strong, and I do not find any evidence supporting it in the results section. Could the authors clarify what they mean by "oppression"? Additionally, the sentence “as gender norms and experiences of masculinities and femininities reflected the division of roles and access to resources by men and women, which in turn shaped their ability to seek early and better healthcare interventions” seems to contradict the notion of oppression if participants are able to seek early and improved healthcare interventions. | The use of the term ‘oppression’ was intended to express the deeply rooted patriarchal and unequal power structures that drive entrenched social and cultural hierarchies between men and women which shape disparities in healthcare access and treatment outcomes. We have, however, revised the sentence by omitting oppression, and the sentence now reads as ‘follows: ‘‘Women’s lived experiences of NCD conditions reflected historical and patriarchal disadvantages………’’ (Please see the revised sentence in lines 31 – 33 in the abstract) |
| 2 | Page 6, line 133: typo: a dot is in the middle of the sentence. Consider removing | Thank you for spotting the typo. It has been addressed |
| 3 | Page 7 on selection of study participants: How did researchers identify participants based on diabetes and hypertension criteria, given that these medical conditions require a confirmed diagnosis from health professionals? Additionally, were any participants identified using a snowball sampling technique? | Participant selection for NCD categories was self-reporting. Since this qualitative study explores narrative accounts of NCDs, we consider self-reporting of illness as appropriate, as it aligns with the aim of the study, which was not to establish clinical prevalence of the conditions reported. Moreover, self-reporting of illness is acceptable and is being used in qualitative studies (Ali et al., 2023; Seckin et al., 2024)). While we recognise the risk of misclassification of disease conditions, this does not affect the quality of the study (please see additions in the text in lines 174 - 177) |
| 4 | It is mentioned that this study was conducted as part of the broad ARISE project. Can the authors clarify whether the study participants were enrolled into the main project and if so, to what extent this initial involvement of the main study had impacted the data collection? | This study was part of a PhD study within the broader [ARISE research consortium](https://www.ariseglobalnetwork.org/ariseannualreport2024) which provided a conceptual understanding of health inequities in informal settlements. While both studies were influenced by the understanding that health inequities are shaped by broader social and structural factors, there were variations in terms of research focus, and participant selection. The wider ARISE study employed multi-stage participatory and mixed methods approaches to understand health inequities from a governance and accountability perspective, while this study explored health inequity from an intersectional lens, drawing on participants’ narratives. The participants were selected independent of the ARISE research. Please find the new additions in lines 143 – 153. |
| 5 | An important section on data collection is missing. I would like authors to include this in order to explain what specific data were collected at each round, why was it necessary to split data collection over different timepoints, where and in which language were data collected, how long lasted interviews on average, and who collected the data, any bias that may result from the way the study participants perceived the data collection team during fieldwork? | The methodology section has been updated, detailing the kinds of data collected at the different stages of the research (155 -169). The sequencing of data collection was essential in understanding the detailed and complex personal narratives of people impacted by NCDs. Collecting data in stages is important in narrative studies as it allows time to build trust, and insights into difficult subjects. Our experience using this kind of approach shows that participants may find it difficult to speak about emotionally sensitive issues (e.g. social and financial disruptions related to ill health, and constrained access to care). Because the interviews were open ended, most of them lasted for over an hour. To ensure consistency and trust, all the interviews were conducted by the lead author (AC). Regarding how participants viewed the research approach, some were initially uncomfortable speaking about their personal lives, as they weren’t convinced about the motives of the research, as it was done in repeated forms (despite being provided with adequate information). However, as the interviews progressed to other stages, participants realised that the interviews may have long term impacts of improving the health care delivery system in informal settlements. |
| 6 | The data analysis section requires further clarification. It would be beneficial if the authors could elaborate on their data coding strategy. It appears that a deductive approach was employed, yet they did not explain how the coding frame was initially developed before its application to the data, nor did they specify who conducted these procedures. Additionally, information regarding the software used to facilitate the data analysis is missing. | We have updated the analysis section explaining the approaches used (193 - 205), which was both inductive and deductive. We applied a framework approach inductively an deductively to analytically observe patterns in the data and to develop new themes. A coding framework was developed based on the study objectives and emerging insights from the data, using NVivo software to further organise and structure the themes. The analysis considered key areas of analysis, including gender, disease conditions and age. |
| 7 | The use of relevant qualitative data reporting guideline is missing. This is important to include as it will help ensure that ensure that the methods and findings are clearly and transparently communicated in the manuscript. | Thank you for suggesting the use of a qualitative reporting guideline, whose aim is to promote research transparency and trustworthiness. Given this, we have tried to maintain transparency by applying the COREQ reporting format to provide additional details about methodological approach, participant selection, data collection, description of research sites, relationship with local community structures (e.g. co-researchers) and analysis of data, using the Nvivo software, coding procedures, design of codes and themes, adaptation of a conceptual framework, and the writing of findings in the methods, participant recruitment, analysis and safeguarding sections. |
| 8 | In relation to adapting the framework for Infectious Diseases of Poverty Research, it would be beneficial if the authors could clarify the specific adaptations made to the framework and how it was applied during data analysis. | The analysis section has been revised with additional texts on the adaptation of the conceptual framework. We adapted the Intersectional Gender Analysis Framework for Infectious Diseases of Poverty Research (WHO, 2020) which was originally adapted from the Intersectionality Wheel (Simpson, 2009). The Intersectional Gender Analysis Framework contains elements such as sex identity and gender norms, to explain how these identities influence disease vulnerability (e.g. poverty, and poor healthcare access), disease severity (e.g. disability), and the limited capacity to seek early and appropriate care. We then adapted the Intersectional Gender Analysis Framework to the study of NCDs in informal settlements by layering the vulnerabilities into macro structural factors (conflict and fragility, urbanisation and health systems) and meso factors (gender norms, poverty, place of living and tenure status), interacting with micro individual circumstances (housing, health, disability and livelihoods) to shape people’s lived experiences of NCDs and access to healthcare. The adaptation of this framework reflects the need to approach NCDs within policy from a bio-social perspective, rather than exclusively from a biomedical approach, which overlooks the underlying social vulnerabilities. |
| 9 | They mentioned that participants who faced distress during the interviews were allowed to grieve and were consoled. How many participants developed such condition during interviews at which round of data collection, and to what extent did this impact on data collected? | The study adapted safeguarding protocols in line with the ARISE research safeguarding policy to protect participants and the research team from harm, including physical and distress. During the research there were only two participants who expressed emotional distress, particularly during the first round of the interviews (line 228 – 235). In these instances, grieving and consolation were observed to be effective, as it helped to build rapport and trust. The grieving impacted positively on the data collection as it allowed these participants to share their lived experiences. While the moments of grieving were intense, it helped to create a shared space built on trust, helping them to accept and live with their health challenges. Requests were made to refer the participants to professional counselling services. However, no referrals were made as participants reported that they were relieved after discussing their challenges with the first author (AC). |
| 10 | This study seeks to explore the lived experiences of individuals affected by NCDs in informal settlements. However, the findings presented in the results section, particularly the initial part titled "lived experience of NCD," fall short of adequately conveying the participants' current experiences with these health conditions. Firstly, the authors include excerpts from three informants but fail to demonstrate how these accounts are representative of all study participants. Secondly, the discussion of informants' lives prior to developing their current health conditions renders the information less pertinent to the study's objectives. It would be beneficial if the authors could focus on participants current lives to discuss the intersectionality of NCDs with social, economic, and gender factors within the participants' living environments, and how these elements contribute to shaping the management of their health conditions. This way of presenting the findings restricts our understanding, as the same observation is reiterated throughout the discussion section. | Thank you for your inquiry about the study not conveying experiences of health seeking. The findings were presented in three sections to provide context about the intersecting challenges impacting NCD outcomes and health seeking decision making which are often less understood by health system policy makers and providers. The first section presents contextual factors (e.g. gender norms, financial and educational barriers and participation in household and healthcare decision making), shaping participants’ reduced capacity to respond to NCD health problems; the second section describes participants’ differential strategies and pathways in coping with NCDs, influenced by support systems, tenure status and knowledge of disease management based on their different identities. The final part links health seeking patterns to the underlying challenges described in the earlier sections. To account for the experiences of structural barriers and health seeking across the three sections, we placed the stories of two participants (male and female in boxes 1 and 2) to illustrate how their experiences cut across different themes and also more broadly represent other participants with similar circumstances, including gender identities and disease conditions.  Your second observation was that participants’ past lives are not pertinent to their lived experiences of NCDs. We respectfully disagree with you on this and argue that the use of a narrative inquiry makes it ideal to connect participants’ past and present realities to make sense of their holistic experiences. In narrative studies, stories form a critical part of the inquiry, which help to present a sequence of events that tie the current to the past. It also demonstrates that people’s lives, their opportunities and challenges faced, in part due to their social status and identity, influence their vulnerability and experiences of illness, which is also part of the intersectional approach. Therefore, excluding past events will mask relevant insights into these historical connections. |
| 11 | In the context of chronic disease, it can be assumed that participants with diabetes, hypertension, or comorbidities might encounter similar yet distinct challenges. It would be beneficial for the authors to discuss the findings across these cases, highlighting both similarities and differences, and to establish a hierarchy within the data. | To illustrate the varying experiences of participants, we have revised the findings and discussion sections as much as possible to present the findings based on gender and disease conditions. Based on your suggestion, we have also conducted further analysis and provided a table (annex 1) that describes the health seeking patterns of participants with different conditions to observe and understand similarities and differences in health seeking patterns (See annex 1). |
| 12 | The use of terms like "people," "women," or "men" in the results section can be misleading, as they might suggest that the findings apply to the general population. Instead, it is advisable to use terms such as "informants," "study participants," or "men" or "women in this study" to ensure that the information reported is limited to those individuals who participated in this data collection. | The use of the term ‘people’ appeared in different places, as you suggested. This was not meant to generalise our findings, but to present the findings in contextually meaningful ways, since this is a narrative study that draws from the experiences of individuals. For example, you may observe that participants in their own narratives often related their experiences to other people living with similar conditions they were living in. However, we have made specific changes to reduce the use of people and instead use ‘participants’ as reflected in lines 248; 318; 323; 467; 585; 684 and 790. You will also notice that we referred to participants as ‘women and men’ in some instances to personalise their experiences related to specific NCD conditions and health seeking in lines 363, 441; 498; 587 and 671 |
