# Supplementary material for: Intersectional experiences of non-communicable diseases and health seeking strategies in informal settlements in Freetown, Sierra Leone
Source: PLOS Glob Public Health. 2026 Jul 1;6(7):e0005263. doi: 10.1371/journal.pgph.0005263 (PMC13322540; doi:10.1371/journal.pgph.0005263)
Supplement: Peer review history

# Peer review: Decision Letter 0

**DOI:** 10.1371/journal.pgph.0005263.r001

**Authors:** Prashanth Nuggehalli Srinivas

27 Oct 2025

PGPH-D-25-02619

Intersectional Experiences of Non-Communicable Diseases and Health Seeking Strategies in Informal Settlements in Freetown, Sierra Leone

PLOS Global Public Health

Dear Dr. Conteh,

Thank you for submitting your manuscript to PLOS Global Public Health. After careful consideration, we feel that it has merit but does not fully meet PLOS Global Public Health’s publication criteria as it currently stands. Therefore, we invite you to submit a revised version of the manuscript that addresses the points raised during the review process.

Please submit your revised manuscript by Dec 11 2025 11:59PM. If you will need more time than this to complete your revisions, please reply to this message or contact the journal office at globalpubhealth@plos.org. When you're ready to submit your revision, log on to https://www.editorialmanager.com/pgph/ and select the 'Submissions Needing Revision' folder to locate your manuscript file.

Please include the following items when submitting your revised manuscript:

- A rebuttal letter that responds to each point raised by the editor and reviewer(s). You should upload this letter as a separate file labeled 'Response to Reviewers'.

- A marked-up copy of your manuscript that highlights changes made to the original version. You should upload this as a separate file labeled 'Revised Manuscript with Track Changes'.

- An unmarked version of your revised paper without tracked changes. You should upload this as a separate file labeled 'Manuscript'.

Guidelines for resubmitting your figure files are available below the reviewer comments at the end of this letter.

We look forward to receiving your revised manuscript.

Kind regards,

Prashanth Nuggehalli Srinivas, MBBS, MPH, PhD

Academic Editor

PLOS Global Public Health

Journal Requirements:

1. Please include a complete copy of PLOS’ questionnaire on inclusivity in global research in your revised manuscript. Our policy for research in this area aims to improve transparency in the reporting of research performed outside of researchers’ own country or community. The policy applies to researchers who have travelled to a different country to conduct research, research with Indigenous populations or their lands, and research on cultural artefacts. The questionnaire can also be requested at the journal’s discretion for any other submissions, even if these conditions are not met.  Please find more information on the policy and a link to download a blank copy of the questionnaire here: https://journals.plos.org/globalpublichealth/s/best-practices-in-research-reporting. Please upload a completed version of your questionnaire as Supporting Information when you resubmit your manuscript.

If the reviewer comments include a recommendation to cite specific previously published works, please review and evaluate these publications to determine whether they are relevant and should be cited. There is no requirement to cite these works unless the editor has indicated otherwise.

Additional Editor Comments (if provided):

[Note: HTML markup is below. Please do not edit.]

Reviewers' comments:

Reviewer's Responses to Questions

**Comments to the Author**

1. Does this manuscript meet PLOS Global Public Health’s publication criteria? Is the manuscript technically sound, and do the data support the conclusions? The manuscript must describe methodologically and ethically rigorous research with conclusions that are appropriately drawn based on the data presented.

Reviewer #1: Yes

Reviewer #2: Yes

**********

2. Has the statistical analysis been performed appropriately and rigorously?

Reviewer #1: N/A

Reviewer #2: N/A

**********

3. Have the authors made all data underlying the findings in their manuscript fully available (please refer to the Data Availability Statement at the start of the manuscript PDF file)?

The PLOS Data policy requires authors to make all data underlying the findings described in their manuscript fully available without restriction, with rare exception. The data should be provided as part of the manuscript or its supporting information, or deposited to a public repository. For example, in addition to summary statistics, the data points behind means, medians and variance measures should be available. If there are restrictions on publicly sharing data—e.g. participant privacy or use of data from a third party—those must be specified.

Reviewer #1: No

Reviewer #2: No

**********

4. Is the manuscript presented in an intelligible fashion and written in standard English?

PLOS Global Public Health does not copyedit accepted manuscripts, so the language in submitted articles must be clear, correct, and unambiguous. Any typographical or grammatical errors should be corrected at revision, so please note any specific errors here.

Reviewer #1: Yes

Reviewer #2: Yes

**********

5. Review Comments to the Author

Please use the space provided to explain your answers to the questions above. You may also include additional comments for the author, including concerns about dual publication, research ethics, or publication ethics. (Please upload your review as an attachment if it exceeds 20,000 characters)

Reviewer #1: The article is relevant and timely. Please use COREQ guidelines/other community guidelines for qualitative research listed on the EQUATOR network to improve the rigour and flow of the article. Additional suggestions for your consideration are listed below.

1. The rationale for the article as well as research question needs to be made more explicit. Additionally, the novelty of the article is relatively unclear.

2. The use of a framework devised for infectious diseases needs to be examined- why was this framework chosen?

3. Line 52-54: It may be useful to examine the cause of death in context of age; additionally, the later use of terms like young-old-middle aged are ambiguous later in the article, please provide an operational definition.

4. Line 54-57: The reason for prioritising lived experience is unclear. The logical flow establishing the rationale for this inquiry needs strengthening.

5. The linkages of NCDs with urbanisation need more citation and can be explored better. The authors may consider reassessing the introduction section which seems to appear definitive without providing a research gap.

6. Why is this research needed? What makes it relevant for global health? This needs to be elucidated in the text.

7. The use of the word “drug peddler” should be in quotes at least at first use, to ensure that it is seen in its local context.

8. The narrative findings are rich and well presented- the global health impact of these findings/future paths based on these findings need to be be made explicit.

Reviewer #2: This manuscript is well-written and engaging, and I would like to commend the authors for presenting such rich and dense information.

Here are my comments, which I believe will enhance the content of this manuscript.

1. Page 2, line 27 (abstract section): The term “oppression” appears quite strong, and I do not find any evidence supporting it in the results section. Could the authors clarify what they mean by "oppression"? Additionally, the sentence “as gender norms and experiences of masculinities and femininities reflected the division of roles and access to resources by men and women, which in turn shaped their ability to seek early and better healthcare interventions” seems to contradict the notion of oppression if participants are able to seek early and improved healthcare interventions.

2. Page 6, line 133: typo: a dot is in the middle of the sentence. Consider removing.

3. Page 7 on selection of study participants: How did researchers identify participants based on diabetes and hypertension criteria, given that these medical conditions require a confirmed diagnosis from health professionals? Additionally, were any participants identified using a snowball sampling technique?

4. It is mentioned that this study was conducted as part of the broad ARISE project. Can the authors clarify whether the study participants were enrolled into the main project and if so, to what extent this initial involvement of the main study had impacted the data collection?

5. An important section on data collection is missing. I would like authors to include this in order to explain what specific data were collected at each round, why was it necessary to split data collection over different timepoints, where and in which language were data collected, how long lasted interviews on average, and who collected the data, any bias that may result from the way the study participants perceived the data collection team during fieldwork?

6. The data analysis section requires further clarification. It would be beneficial if the authors could elaborate on their data coding strategy. It appears that a deductive approach was employed, yet they did not explain how the coding frame was initially developed before its application to the data, nor did they specify who conducted these procedures. Additionally, information regarding the software used to facilitate the data analysis is missing.

7. The use of relevant qualitative data reporting guideline is missing. This is important to include as it will help ensure that ensure that the methods and findings are clearly and transparently communicated in the manuscript.

8. In relation to adapting the framework for Infectious Diseases of Poverty Research, it would be beneficial if the authors could clarify the specific adaptations made to the framework and how it was applied during data analysis.

9. They mentioned that participants who faced distress during the interviews were allowed to grieve and were consoled. How many participants developed such condition during interviews at which round of data collection, and to what extent did this impact on data collected?

10. This study seeks to explore the lived experiences of individuals affected by NCDs in informal settlements. However, the findings presented in the results section, particularly the initial part titled "lived experience of NCD," fall short of adequately conveying the participants' current experiences with these health conditions. Firstly, the authors include excerpts from three informants but fail to demonstrate how these accounts are representative of all study participants. Secondly, the discussion of informants' lives prior to developing their current health conditions renders the information less pertinent to the study's objectives. It would be beneficial if the authors could focus on participants current lives to discuss the intersectionality of NCDs with social, economic, and gender factors within the participants' living environments, and how these elements contribute to shaping the management of their health conditions. This way of presenting the findings restricts our understanding, as the same observation is reiterated throughout the discussion section.

11. In the context of chronic disease, it can be assumed that participants with diabetes, hypertension, or comorbidities might encounter similar yet distinct challenges. It would be beneficial for the authors to discuss the findings across these cases, highlighting both similarities and differences, and to establish a hierarchy within the data.

12. The use of terms like "people," "women," or "men" in the results section can be misleading, as they might suggest that the findings apply to the general population. Instead, it is advisable to use terms such as "informants," "study participants," or "men" or "women in this study" to ensure that the information reported is limited to those individuals who participated in this data collection.

**********

6. PLOS authors have the option to publish the peer review history of their article (what does this mean?). If published, this will include your full peer review and any attached files.

**Do you want your identity to be public for this peer review?**  If you choose “no”, your identity will remain anonymous but your review may still be made public.

For information about this choice, including consent withdrawal, please see our Privacy Policy.

Reviewer #1: **Yes:** Shivangi Shankar

Reviewer #2: No

**********

[NOTE: If reviewer comments were submitted as an attachment file, they will be attached to this email and accessible via the submission site. Please log into your account, locate the manuscript record, and check for the action link "View Attachments". If this link does not appear, there are no attachment files.]

-->Figure Resubmissions:

While revising your submission, we strongly recommend that you use PLOS’s NAAS tool (https://ngplosjournals.pagemajik.ai/artanalysis) to test your figure files. NAAS can convert your figure files to the TIFF file type and meet basic requirements (such as print size, resolution), or provide you with a report on issues that do not meet our requirements and that NAAS cannot fix.

After uploading your figures to PLOS’s NAAS tool - https://ngplosjournals.pagemajik.ai/artanalysis, NAAS will process the files provided and display the results in the "Uploaded Files" section of the page as the processing is complete. If the uploaded figures meet our requirements (or NAAS is able to fix the files to meet our requirements), the figure will be marked as "fixed" above. If NAAS is unable to fix the files, a red "failed" label will appear above. When NAAS has confirmed that the figure files meet our requirements, please download the file via the download option, and include these NAAS processed figure files when submitting your revised manuscript.

Attachment Submitted filename: Review assignment for PGPH-D-25-02619.docx

# Author comment: Author response to Decision Letter 1

**DOI:** 10.1371/journal.pgph.0005263.r002

24 Feb 2026

Attachment Submitted filename: Response to Reviewers_PLOS_31_01_26.docx

# Peer review: Decision Letter 1

**DOI:** 10.1371/journal.pgph.0005263.r003

**Authors:** Ilse Bloom

30 Apr 2026

PGPH-D-25-02619R1

Intersectional Experiences of Non-Communicable Diseases and Health Seeking Strategies in Informal Settlements in Freetown, Sierra Leone

PLOS Global Public Health

Dear Dr. Conteh,

Thank you for submitting your manuscript to PLOS Global Public Health. After careful consideration, we feel that it has merit but does not fully meet PLOS Global Public Health’s publication criteria as it currently stands. Therefore, we invite you to submit a revised version of the manuscript that addresses the points raised during the review process.

The manuscript has been further evaluated by one reviewer, and their comments are available below.

We would also like to request that the authors attach the following:

- The COREQ checklist; (http://journals.plos.org/globalpublichealth/s/submission-guidelines#loc-qualitative-research).

- The final interview guide.

Could you please carefully revise the manuscript to address all comments raised?

Please submit your revised manuscript by May 30 2026 11:59PM. If you will need more time than this to complete your revisions, please reply to this message or contact the journal office at globalpubhealth@plos.org. When you're ready to submit your revision, log on to https://www.editorialmanager.com/pgph/ and select the 'Submissions Needing Revision' folder to locate your manuscript file.

Please include the following items when submitting your revised manuscript:

- A letter that responds to each point raised by the editor and reviewer(s). You should upload this letter as a separate file labeled 'Response to Reviewers'.

- A marked-up copy of your manuscript that highlights changes made to the original version. You should upload this as a separate file labeled 'Revised Manuscript with Track Changes'.

- An unmarked version of your revised paper without tracked changes. You should upload this as a separate file labeled 'Manuscript'.

Guidelines for resubmitting your figure files are available below the reviewer comments at the end of this letter.

As the corresponding author, your ORCID iD is verified in the submission system and will appear in the published article. PLOS supports the use of ORCID, and we encourage all coauthors to register for an ORCID iD and use it as well. Please encourage your coauthors to verify their ORCID iD within the submission system before final acceptance, as unverified ORCID iDs will not appear in the published article. *Only*  the individual author can complete the verification step; PLOS staff *cannot*  verify ORCID iDs on behalf of authors.

We look forward to receiving your revised manuscript.

Kind regards,

Ilse Bloom

Staff Editor

PLOS Global Public Health

Journal Requirements:

1. Please update your online Competing Interests statement. If you have no competing interests to declare, please state: "The authors have declared that no competing interests exist."

2. If the reviewer comments include a recommendation to cite specific previously published works, please review and evaluate these publications to determine whether they are relevant and should be cited. There is no requirement to cite these works unless the editor has indicated otherwise.

3. Please review your reference list to ensure that it is complete and correct. If you have cited papers that have been retracted, please include the rationale for doing so in the manuscript text, or remove these references and replace them with relevant current references. Any changes to the reference list should be mentioned in the rebuttal letter that accompanies your revised manuscript. If you need to cite a retracted article, indicate the article’s retracted status in the References list and also include a citation and full reference for the retraction notice.

Additional Editor Comments (if provided):

[Note: HTML markup is below. Please do not edit.]

Reviewers' comments:

Reviewer's Responses to Questions

**Comments to the Author**

1. If the authors have adequately addressed your comments raised in a previous round of review and you feel that this manuscript is now acceptable for publication, you may indicate that here to bypass the “Comments to the Author” section, enter your conflict of interest statement in the “Confidential to Editor” section, and submit your "Accept" recommendation.

Reviewer #1: All comments have been addressed

**********

2. Does this manuscript meet PLOS Global Public Health’s publication criteria? Is the manuscript technically sound, and do the data support the conclusions? The manuscript must describe methodologically and ethically rigorous research with conclusions that are appropriately drawn based on the data presented.

Reviewer #1: Yes

**********

3. Has the statistical analysis been performed appropriately and rigorously?

Reviewer #1: N/A

**********

4. Have the authors made all data underlying the findings in their manuscript fully available (please refer to the Data Availability Statement at the start of the manuscript PDF file)?

The PLOS Data policy requires authors to make all data underlying the findings described in their manuscript fully available without restriction, with rare exception. The data should be provided as part of the manuscript or its supporting information, or deposited to a public repository. For example, in addition to summary statistics, the data points behind means, medians and variance measures should be available. If there are restrictions on publicly sharing data—e.g. participant privacy or use of data from a third party—those must be specified.

Reviewer #1: Yes

**********

5. Is the manuscript presented in an intelligible fashion and written in standard English?

PLOS Global Public Health does not copyedit accepted manuscripts, so the language in submitted articles must be clear, correct, and unambiguous. Any typographical or grammatical errors should be corrected at revision, so please note any specific errors here.

Reviewer #1: Yes

**********

6. Review Comments to the Author

Please use the space provided to explain your answers to the questions above. You may also include additional comments for the author, including concerns about dual publication, research ethics, or publication ethics. (Please upload your review as an attachment if it exceeds 20,000 characters)

Reviewer #1: All suggestions have been addressed thoughtfully and meaningfully. The article is important and timely for presenting lived experiences with NCDs.

**********

7. PLOS authors have the option to publish the peer review history of their article (what does this mean?). If published, this will include your full peer review and any attached files.

**Do you want your identity to be public for this peer review?**  If you choose “no”, your identity will remain anonymous but your review may still be made public.

For information about this choice, including consent withdrawal, please see our Privacy Policy.

Reviewer #1: **Yes:** Shivangi Shankar

**********

[NOTE: If reviewer comments were submitted as an attachment file, they will be attached to this email and accessible via the submission site. Please log into your account, locate the manuscript record, and check for the action link "View Attachments". If this link does not appear, there are no attachment files.]

Figure Resubmissions:

While revising your submission, we strongly recommend that you use PLOS’s NAAS tool (https://ngplosjournals.pagemajik.ai/artanalysis) to test your figure files. NAAS can convert your figure files to the TIFF file type and meet basic requirements (such as print size, resolution), or provide you with a report on issues that do not meet our requirements and that NAAS cannot fix.

After uploading your figures to PLOS’s NAAS tool - https://ngplosjournals.pagemajik.ai/artanalysis, NAAS will process the files provided and display the results in the "Uploaded Files" section of the page as the processing is complete. If the uploaded figures meet our requirements (or NAAS is able to fix the files to meet our requirements), the figure will be marked as "fixed" above. If NAAS is unable to fix the files, a red "failed" label will appear above. When NAAS has confirmed that the figure files meet our requirements, please download the file via the download option, and include these NAAS processed figure files when submitting your revised manuscript.

# Author comment: Author response to Decision Letter 2

**DOI:** 10.1371/journal.pgph.0005263.r004

21 May 2026

Attachment Submitted filename: Response_to_Reviewers_PLOS_31_01_26_auresp_2.docx

# Editor report: Decision Letter 2

**DOI:** 10.1371/journal.pgph.0005263.r005

**Authors:** Julia Robinson

28 May 2026

Intersectional Experiences of Non-Communicable Diseases and Health Seeking Strategies in Informal Settlements in Freetown, Sierra Leone

PGPH-D-25-02619R2

Dear Urban Health Researcher Conteh,

We are pleased to inform you that your manuscript 'Intersectional Experiences of Non-Communicable Diseases and Health Seeking Strategies in Informal Settlements in Freetown, Sierra Leone' has been provisionally accepted for publication in PLOS Global Public Health.

Before your manuscript can be formally accepted you will need to complete some formatting changes, which you will receive in a follow up email. A member of our team will be in touch with a set of requests.

Please note that your manuscript will not be scheduled for publication until you have made the required changes, so a swift response is appreciated.

IMPORTANT: The editorial review process is now complete. PLOS will only permit corrections to spelling, formatting or significant scientific errors from this point onwards. Requests for major changes, or any which affect the scientific understanding of your work, will cause delays to the publication date of your manuscript.

If your institution or institutions have a press office, please notify them about your upcoming paper to help maximize its impact. If they'll be preparing press materials, please inform our press team as soon as possible -- no later than 48 hours after receiving the formal acceptance. Your manuscript will remain under strict press embargo until 2 pm Eastern Time on the date of publication. For more information, please contact globalpubhealth@plos.org.

Thank you again for supporting Open Access publishing; we are looking forward to publishing your work in PLOS Global Public Health.

Best regards,

Julia Robinson

Executive Editor

PLOS Global Public Health

***********************************************************

Reviewer Comments (if any, and for reference):
